# Effectiveness of Interventions for Managing Acute Malnutrition in Children under Five Years of Age in Low-Income and Middle-Income Countries: A Systematic Review and Meta-Analysis

**DOI:** 10.3390/nu12010116

**Published:** 2020-01-01

**Authors:** Jai K. Das, Rehana A. Salam, Marwah Saeed, Faheem Ali Kazmi, Zulfiqar A. Bhutta

**Affiliations:** 1 Division of Women and Child Health, Aga Khan University Hospital, Karachi 74800, Pakistan; rehana.salam@aku.edu (J.K.D.); jai.das@aku.edu (R.A.S.); sfak1992@gmail.com (F.A.K.); 2 Medical Student, Aga Khan University, Karachi 74800, Pakistan; marwah.m508273@student.aku.edu; 3 Centre of Excellence in Women and Child Health, Aga Khan University, Karachi 74800, Pakistan; 4 Centre for Global Child Health, the Hospital for Sick Children, Toronto, ON M5G 0A4, Canada

**Keywords:** malnutrition, children, acute malnutrition

## Abstract

Childhood malnutrition is a major public health concern, as it is associated with significant short- and long-term morbidity and mortality. The objective of this review was to comprehensively review the evidence for the management of severe acute malnutrition (SAM) and moderate acute malnutrition (MAM) according to the current World Health Organization (WHO) protocol using facility- and community-based approaches, as well as the effectiveness of ready-to-use therapeutic food (RUTF), ready-to-use supplementary food (RUSF), prophylactic antibiotic use, and vitamin A supplementation. We searched relevant electronic databases until 11 February 2019, and performed a meta-analysis. This review summarizes findings from a total of 42 studies (48 papers), including 35,017 children. Limited data show some benefit of integrated community-based screening, identification, and management of SAM and MAM on improving recovery rate. Facility-based screening and management of uncomplicated SAM has no effect on recovery and mortality, while the effect of therapeutic milk F100 for SAM is comparable to RUTF for weight gain and mortality. Local food and whey RUSF are comparable to standard RUSF for recovery rate and weight gain in MAM, while standard RUSF has additional benefits to CSB. Prophylactic antibiotic administration in uncomplicated SAM improves recovery rate and probably improves weight gain and reduces mortality. Limited data suggest that high-dose vitamin A supplementation is comparable with low-dose vitamin A supplementation for weight gain and mortality among children with SAM.

## 1. Introduction

Childhood undernutrition includes wasting (weight-for-height z-score (WHZ) < −2SD), stunting (height-for-age z-score (HAZ) < −2SD), underweight (weight-for-age z-score (WAZ) < −2SD), and micronutrient deficiencies or insufficiencies [1]. The current World Health Organization (WHO) guidelines subsume these entities into the blanket term of childhood malnutrition, which is broadly categorized into acute and chronic malnutrition. Acute malnutrition is further classified on the basis of severity into moderate acute malnutrition (MAM) (WHZ between −3 and −2) and severe acute malnutrition (SAM) (WHZ < −3 and mid-upper arm circumference (MUAC) < 115 mm), whereas chronic malnutrition occurs due to long-term insufficient intake of nutrients and a complex interplay of intergenerational and environmental factors, resulting in stunting [2]. In 2017, an estimated 155 million children under five years of age were stunted and 52 million were wasted [3]. Asia and Africa still share the greatest burden of malnutrition, with more than half of all stunted children and two-thirds of all wasted children under five years of age living in Asia, and over one-third of stunted children and a quarter of wasted children living in Africa [4]. In Asia and Oceania, nearly 10% of children under five years of age are at increased risk of death due to wasting [4].

Childhood malnutrition is a major public health concern, since it is associated with significant morbidity and mortality [1]. The consequences of malnutrition among infants and children can be short-term, such as morbidity, mortality, and disability; or long-term, including impaired cognitive development, increased risk of disease due to either concurrent infections or metabolic disorders, and suboptimal economic productivity [5]. Undernutrition, including stunting, severe wasting, deficiencies of vitamin A and zinc, and suboptimal breastfeeding, has been an underlying cause of approximately one-third of the mortality among children under five years of age [4,5]. Childhood malnutrition is a result of a complex interplay of nutrition-specific and nutrition-sensitive factors. Nutrition-specific factors include inadequate food and nutrient intake, poor feeding, caregiving, and parenting practices, and burden of infectious diseases. Nutrition-sensitive factors include food insecurity; inadequate caregiving resources at the maternal, household, and community levels; limited access to health services; and unhygienic environment [6]. Improving childhood malnutrition requires effective implementation of nutrition-sensitive as well as nutrition-specific interventions [7].

Despite the outlined interventions to manage childhood malnutrition [8], there is uncertainty around the most effective methods to treat malnutrition in young children [9]. The existing WHO guidelines for the management of malnutrition also highlighted a few priority issues and research gaps [8] pertaining to strategies to improve active community screening; clinical effect and cost effectiveness of giving prophylactics oral antibiotics; adverse effects of giving broad-spectrum antibiotics; efficacy and effectiveness of different ready to use supplementary food (RUSF) and ready-to-use therapeutic foods (RUTF); and efficacy of daily low-dose vitamin A supplementation compared to single high-dose vitamin A. The above research gaps from the WHO guidelines have not been the topic of a comprehensive systematic review. However, there are a few existing reviews evaluating certain interventions separately. A systematic review evaluated the effectiveness of approaches to managing MAM and SAM according to the WHO protocol, but the results were unclear due to lack of robust trials [10]. Existing reviews on management of acute malnutrition are either focused on specific population groups; specific interventions (prophylactic use of antibiotics, IV fluid for shock, treatment of diarrhea, micronutrients deficiencies, etc.); or there is discrepancy in the definition of undernutrition and types of therapeutic or supplementary foods [9,11,12,13,14]. Moreover, supplementary feeding has been the topic of two reviews [15,16] and the effectiveness of vitamin A supplementation for the treatment of SAM has also been reviewed [17]. However, there is a need to comprehensively review the current evidence for the effectiveness of various community- and facility-based strategies to identify and manage MAM and SAM, including the community-based screening, identification management of SAM and MAM, relative effectiveness of RUTF for SAM and RUSF for MAM, effectiveness of prophylactic use of antibiotic to manage uncomplicated SAM, and the effectiveness of vitamin A supplementation to manage children with acute malnutrition.

The protocol for this review is published with the Campbell Collaboration at https://onlinelibrary.wiley.com/doi/full/10.1002/CL2.193.

## 2. Materials and Methods

### 2.1. Objective

The objectives of this review are:

- To evaluate the effectiveness of community-based strategies, such as community-based mobilization, screening, follow-up, counselling, and education; to improve screening, identification, and management of SAM and MAM;
- To evaluate the effectiveness of facility-based strategies, such as facility-based screening, management, and periodic follow-up, to improve screening and management of SAM and MAM;
- To evaluate the effectiveness and relative effectiveness of various RUTF and RUSF for the management of SAM and MAM;
- To evaluate the effectiveness of prophylactic use of antibiotic to manage uncomplicated SAM;
- To evaluate the effectiveness of various doses of vitamin A supplement to manage children with SAM and MAM.

### 2.2. Type of Studies and Participants

We included primary studies, including large-scale program evaluations, using experimental and quasi-experimental study designs that allow for causal inference. We included randomized controlled trials (RCTs), including both cluster and individual level randomization, quasi-experimental studies with non-random assignment to intervention and comparison groups, controlled before–after studies (CBA), and interrupted time series (ITS). We included studies targeting children under 5 years of age with SAM and MAM in low- and middle-income countries (LMIC). We used the following definition of MAM and SAM by WHO:

SAM: weight-for-height z-score (WHZ) < −3 SD, weight-for-height (WFH) < 70% of the median National Center for Health Statistics (NCHS) or WHO reference, or mid-upper arm circumference (MUAC) < 115 mm or edema.

Complicated SAM: SAM cases without appetite or with medical complications.

Uncomplicated SAM: SAM children with successful standard appetite test, and without fever, clinical infections, or complications.

MAM: weight-for-height z-score (WHZ) between −2 and −3 standard deviations (SD), WFH equal to 70–80% of the NCHS or WHO reference median, or mid-upper arm circumference (MUAC) of 115–125 mm.

We excluded studies specifically conducted on HIV populations.

### 2.3. Type of Interventions

The following interventions were considered and compared against the suggested comparison groups separately:

Community-based strategies to screen, identify, and manage SAM and MAM compared to non-community-based strategies (e.g., active community-based surveillance by community health workers (CHWs) versus no active surveillance; training of CHWs for community-based screening versus no training; community-based management with RUTF versus standard care practices).

Facility-based strategies to screen and manage uncomplicated SAM according to the WHO protocol compared to other standards of care (e.g., treatment for uncomplicated SAM in health facilities alone versus by CHWs and health facilities; training of health facility staff to diagnose and treat uncomplicated SAM versus no training; facility-based management of SAM according to the WHO protocol versus other adapted protocols).

Community-based management of children with uncomplicated SAM as outpatients with RUTF compared to standard diet, fortified blended flours (FBFs), or other locally produced foods.

RUSF for MAM compared to standard diet, FBF, or other locally produced foods.

Prophylactic use of antibiotics in children with uncomplicated SAM compared to no antibiotics.

Vitamin A supplementation in the management of SAM and MAM with various doses and frequency of administration.

### 2.4. Type of Outcomes

We included studies that met our inclusion criteria, but only included studies in the analysis that reported on the predefined primary outcomes: recovery rate (measured as the number of malnourished children recovered divided by the total number of malnourished children), weight gain (measured as grams/kg/day), relapse (measured as the proportion of children who re-enrolled after they had recovered at any time point reported by study authors), mortality (measured as the proportion of children dying under five years of age), case fatality rates (measured as proportion of malnourished children dying divided by the total malnourished children). The secondary outcomes included height gain, MUAC gain, time to recover (measured as length of time between admission and discharge), stunting (defined as below minus two standard deviations from median height for age of reference population), wasting (defined as below minus two standard deviations from median weight for height of reference population), underweight (defined as below minus two standard deviations from median weight for age of reference population), infection incidence (bacteremia, sepsis, pneumonia, urinary tract infections, meningitis, and diarrhea), adverse effects (such as side effects associated with antibiotics, drug resistance, rapid weight gain, micronutrient toxicity, etc.), hospitalization costs, and cost-effectiveness.

### 2.5. Search Methods

We searched the following databases until 11 February 2019: Cochrane Database of Systematic Reviews (CDSR) and the Cochrane Central Register of Controlled Trials (CENTRAL) in the Cochrane Library; World Health Organization regional databases; The Campbell Library; MEDLINE (PubMed); EMBASE; CINAHL; Web of Science; POPLINE; CAB abstracts and Global Health; PAHO; IndMED (indmed.nic.in/indmed.html); and WHO Global Health Index. We also searched the WHO International Clinical Trials Registry Platform (ICTRP; http://www.who.int/ictrp/en/), ClinicalTrials.gov, and Epistemonikos (https://www.epistemonikos.org). We did not restrict our searches by date, language, or publication status. Search strategy available as Appendix A.

### 2.6. Data Collection and Analysis

Two reviewers screened titles and abstracts in duplicate. We used the PRISMA flow diagram to report eligibility of studies. We retrieved the full text of all studies that passed this first level screening. The full text review were also done in duplicate by two reviewers, and agreement was reached by consensus. Disagreements were resolved by consultation with a third reviewer. We collated multiple reports of the same study, so that each study rather than each report was the unit of interest in the review. We extracted data from each study on study background, population and setting, intervention group details, comparison group details, outcomes, and other information. We performed a statistical meta-analysis using RevMan 5 [18]. For dichotomous data, we used odds ratios (OR) and risk ratios (RR) with 95% confidence intervals (CI). For continuous data, we used the mean difference (MD) with 95% CI if outcomes were measured in the same way between trials. We used the standardized mean difference (SMD) with 95% CI to combine trials that measured the same outcome but used different units or scales of measurement. We used random-effects meta-analysis to combine data to produce an overall summary, since we expected reasonable methodological heterogeneity in interventions, comparisons, outcomes, and settings within the studies included.

Statistical heterogeneity was assessed using Tau^2^, I^2^, and significance of the Chi-square test; we also assessed heterogeneity visually using forest plots. Based on prior theory and clinical knowledge, we expected clinical and methodological heterogeneity in effect sizes in this literature. Depending on data availability, we planned conduct exploratory subgroup analyses for the following subgroups:

Age (1–6 months, 6–59 months);

Duration of intervention (short-term (<3 months), medium-term (3–6 months), and long-term (6–12 months));

Various formulations of supplementary foods;

Setting (Community management, primary care management, and facility management);

Vitamin A supplementation dosage (different doses);

Different antibiotics;

Equity (low income and disadvantaged groups versus relatively high income groups).

We planned to use the Chi2 test to assess subgroup differences. Due to the limited number of studies, we could not conduct the planned subgroup analysis; however, we did separately analyze the various supplementary foods that were compared with standard RUTF and standard RUSF.

### 2.7. Quality Assessment

For RCTs, we used the Cochrane risk of bias tool [19], which assesses selection bias, performance bias, detection bias, attrition bias, and reporting bias. We rated each component as “high”, “low”, or “unclear” for each risk of bias component. For non-randomized studies, we used the Cochrane effective practice and organization of care (EPOC) risk of bias criteria (based on additional criteria, including similar baseline outcome measurements, similar baseline characteristics, knowledge of the allocated interventions adequately prevented during the study, protection against contamination, intervention independent of other changes, shape of intervention effect pre-specified, and intervention unlikely to affect data collection) and rated the studies as low risk, high risk, or unclear risk [20]. We provided supporting evidence for the risk of bias judgements. Two independent reviewers performed quality appraisal for each study and disagreements were resolved by discussion or consultation with a third reviewer.

We planned to conduct sensitivity analysis based on the risk of bias of the included studies by removing studies judged to be at high risk of bias for sequence generation, allocation concealment, and blinding of participants from the meta-analysis to determine whether the removal of studies with high risk of bias impacts the estimates.

We summarized the quality of evidence according to the outcomes as per the grading of recommendations, assessment, development, and evaluation (GRADE) criteria [21]. Grades of “high”, “moderate”, “low”, and “very low” were used to grade the overall evidence, indicating the strength of an effect on a specific health outcome based on methodological flaws within the component studies, consistency of results across different studies, generalizability of research results to the wider patient base, and how effective the treatments were shown to be [22]. For non-randomized studies, the evidence quality was upgraded based on magnitude of effect, dose–response relationship, and the likelihood of all plausible confounding factors to reduce the effect (where an effect was observed) or suggest a spurious effect (when no effect was observed). Two reviewers discussed ratings and reached consensus, and disagreements were resolved by consulting a third reviewer.

## 3. Results

### 3.1. Results of the Search

**Figure 1:**
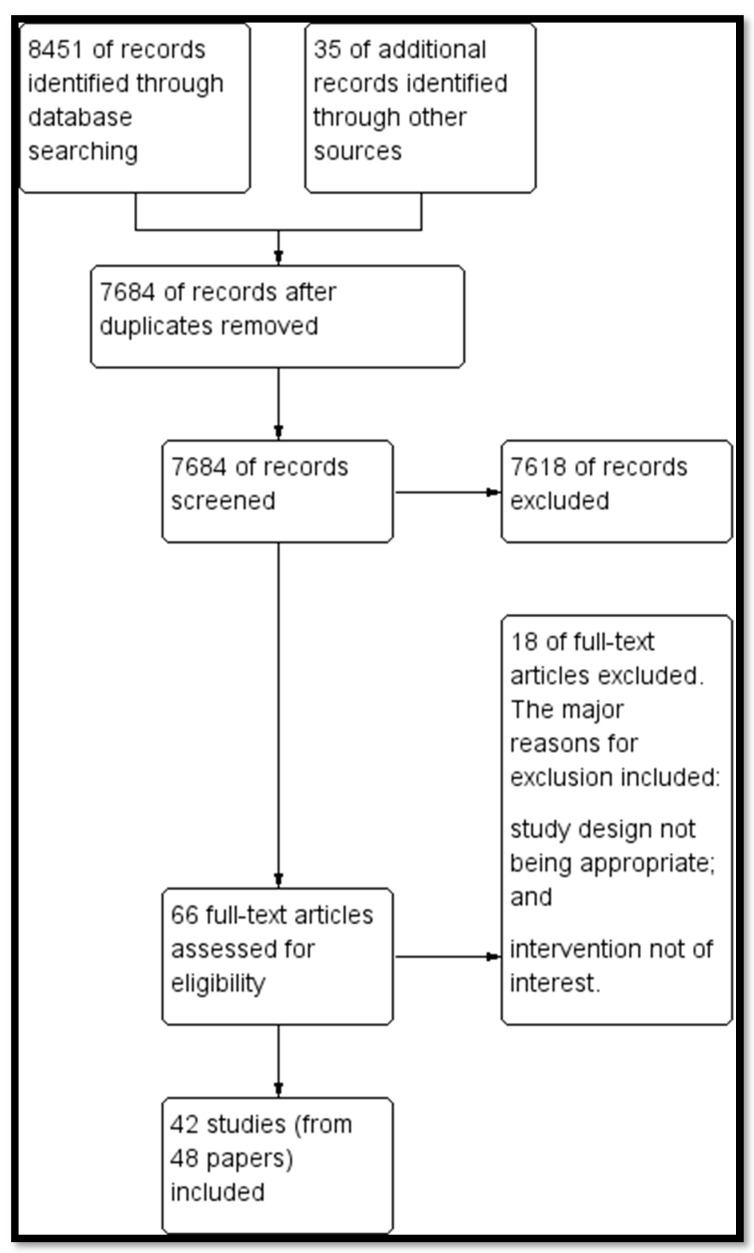
Search Flow Diagram.

Our search identified a total of 8451 potentially relevant titles from the electronic searches and 35 records from searching other sources. After removing duplicates, we screened 7684 records for eligibility and excluded 7618 on the basis of title and abstract. We obtained the full-text reports of the remaining 66 records, and of these, excluded 18 studies and included 42 studies (48 papers). Figure 1 depicts the search flow diagram and the reasons for exclusion for the excluded studies are reported in Appendix B.

### 3.2. Description of Included Studies

We included a total of 42 studies (from 48 papers), including 35,017 children [17,23,24,25,26,27,28,29,30,31,32,33,34,35,36,37,38,39,40,41,42,43,44,45,46,47,48,49,50,51,52,53,54,55,56,57,58,59,60,61,62,63]. Of these, 33 of the studies were RCTs, 6 studies were quasi-experimental studies, and 3 of the studies were cost-effectiveness studies. Four of the included RCTs were cluster RCTs [23,37,40,44], while others were individually randomized trials. All the studies were conducted in either community, hospital, health center, or nutrition rehabilitation centers settings in LMICs, including Bangladesh, Mali, Malawi, Congo, Kenya, India, Niger, Senegal, Sudan, Burkina Faso, Zambia, Ethiopia, Sierra Leonne, Cameroon, Indonesia, and Cambodia. Almost all the included studies targeted children aged six months to 60 months, with a few exceptions: one study [60] targeted children 6–15 months of age, one study [49] targeted children 6–18 months of age, two studies [34,47] targeted children 6–23 months of age, one study [30] targeted children 5–28 months of age, and two studies [23,32] targeted children 6–36 months of age.

Two studies [44,63] assessed community-based strategies: one study compared an integrated community-based protocol to manage MAM and SAM with no community-based strategies, while the other one compared the cost-effectiveness of existing health services with CMAM to the existing health services without CMAM. Seven studies [24,30,35,50,57,59,62] assessed facility-based strategies compared to other standards of care. Three studies [24,30,50] assessed cost-effectiveness of inpatient rehabilitation compared to outpatient or community-based management. Fourteen studies [25,26,27,29,31,32,36,37,39,42,48,51,54,55] compared community-based management of children with uncomplicated SAM with RUTF versus other foods. Other foods included non-dairy or reduced dairy-based RUTF, non-peanut butter-based RUTF, energy dense homemade food, corn soy blend (CSB), and F100. Fourteen studies [23,34,40,41,43,45,46,47,49,53,56,58,61] compared RUSF for MAM with other foods. Other foods included non-dairy or reduced dairy-based RUTF, non-peanut butter-based RUTF, energy dense homemade food, corn soy blend (CSB), and F100. Three studies [17,28,38] compared prophylactic use of antibiotics in children with uncomplicated SAM to no antibiotics. Two studies [33,52] compared high dose vitamin A supplement with low dose vitamin A supplement.

Primary outcomes included recovery rate, weight gain, relapse, and mortality. None of the included studies reported case fatality rates. Among secondary outcome, included studies reported height gain, MUAC gain, time-to-recovery, stunting, wasting, underweight, adverse events, hospitalization, and cost effectiveness. The characteristics of the included studies are specified in Table 1.

### 3.3. Risk of Bias

**Figure 2:**
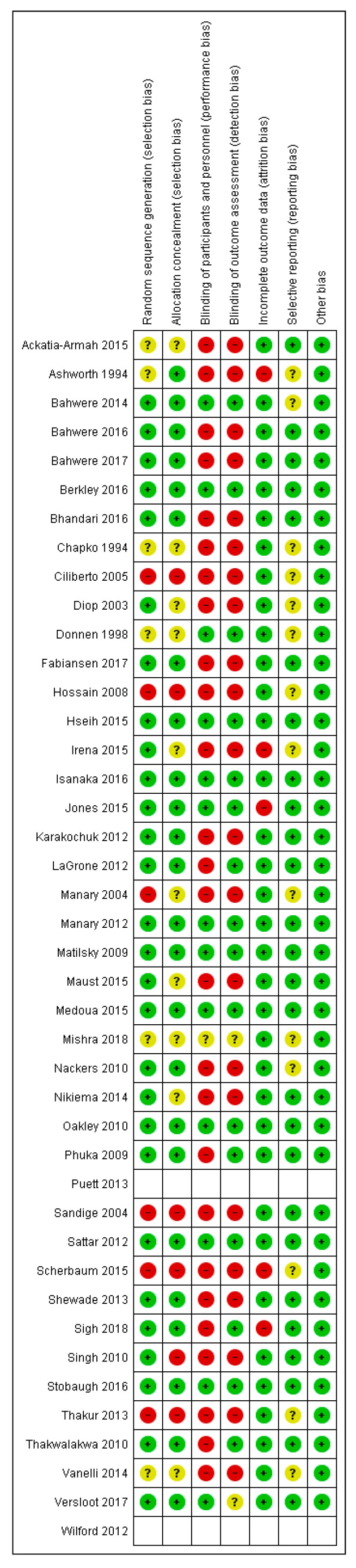
Risk of bias summary. Note: red = high risk; green = low risk; yellow = unclear risk.

All the studies (except two studies [50,63]) were either RCTs or quasi-experimental studies and were assessed for risk of bias using the Cochrane risk of bias tool. Two studies [50,63] were cost-effectiveness studies. Overall, the studies were judged to be at high risk of bias for blinding of participants and personnel and outcome assessment blinding. The summary of the risk of bias across the included studies is shown in Figure 2.

### 3.4. Effects of Intervention

Comparison 1: Community-based strategies to screen, identify, and manage SAM and MAM compared to non-community-based strategies.

Two studies [44,63] assessed community-based strategies. One study [44] compared an integrated community-based protocol to manage MAM and SAM with non-community-based management, which comprised non-community-based surveillance, while one study [63] compared the cost-effectiveness of existing health services with CMAM to the existing health services without CMAM. We could not conduct a meta-analysis for this comparison. Among primary outcomes, integrated community-based management probably improves recovery by 4% (RR: 1.04; 95% CI: 1.00 to 1.09; one study; 1957 participants; moderate quality outcome), decreases weight gain by 0.8 g/kg/day compared to the standard management (MD: −0.80 g/kg/day; 95% CI: −0.82 to −0.78; one study; 1957 participants; moderate quality outcome), while mortality was similar between the two groups (RR: 0.93; 95% CI: 0.60 to 1.45; one study; 1957 participants; moderate outcome quality).

Among secondary outcomes, integrated community-based management probably reduced length gain by 0.1 mm/day compared to standard management (MD: −0.10 mm/day; 95% CI: −0.10 to −0.10; one study; 1957 participants; moderate quality outcome) and probably improved MUAC by 0.27 mm/day compared to the standard management (MD: 0.27 mm/day; 95% CI: 0.27 to 0.27; one study; 1957 participants; moderate quality outcome). One study [44] reported diarrhea and fever as adverse events suggesting that the integrated community-based management probably reduces diarrhea by 29% (RR: 0.71; 95% CI: 0.60 to 0.85; one study; 1957 participants; moderate quality outcome) and fever by 15% (RR: 0.85; 95% CI: 0.77 to 0.93; one study; 1957 participants; moderate quality outcome) compared to the standard management during the first two weeks of feeding.

Two studies [44,63] reported on cost and cost-effectiveness. One study [44] reported that the cost of RUTF used to treat a SAM case in integrated community-based management was $36, whereas for the no community-based management group was $68; the cost of supplementary food used to treat a case of MAM in either the integrated or the standard management group was $12. The study did not report a comparison of the cost-effectiveness of the two management strategies because the costs of care were not documented. The other study [63] assessed the cost-effectiveness of the existing health services with CMAM compared to the existing health services without CMAM, and reported that the CMAM was highly cost-effective in Malawi; however, the study recommended that several contextual and programmatic factors should be considered when generalizing for diverse contexts.

The forest plots for this comparison are provided in the supplementary file (Figures S1–S6).

Comparison 2: Facility-based strategies to screen and manage uncomplicated SAM according to the WHO protocol compared to other standards of care.

Four studies [24,30,35,50] assessed facility-based strategies compared to other standards of care, namely outpatient and community-based management for uncomplicated SAM. Two studies [24,30] were conducted before the current differentiation of complicated and uncomplicated SAM. Among primary outcomes, one study reported recovery at 4–6 weeks, suggesting no evidence of difference on recovery (RR: 1.00; 95% CI: 0.80, 1.25; one study; 60 participants; very low quality evidence). Two studies reported mortality at 4–6 weeks and found no difference of effect on mortality (RR: 1.21; 95% CI: 0.75, 1.94; two studies; 473 participants; I2: 0%; low quality evidence). Among secondary outcomes, included studies only reported cost-effectiveness. One study [24] reported the cost-effectiveness of three approaches (inpatient, daycare, or domiciliary care after one week of daycare) for the management of severely malnourished children. Findings suggest that the average institutional costs to achieve 80% weight-for-height was $156 for the inpatient, $59 for daycare, and $29 for domiciliary care. The study reported that domiciliary care after one week of daycare was the most cost-effective treatment option. One study [30] compared costs between patients assigned to hospital rehabilitation with ambulatory care, with findings suggesting that children assigned to inpatient rehabilitation received significantly more days of hospital care and fewer days of ambulatory care when compared to patients assigned to ambulatory rehabilitation. Moreover, the study reported that the total cost of rehabilitation was significantly higher for hospital rehabilitation. One study [50] assessed the cost-effectiveness of adding CMAM to a community-based health and nutrition program delivered by CHWs in southern Bangladesh. The cost-effectiveness of this model of treatment for SAM was compared with the cost-effectiveness of the “standard of care” for SAM (i.e., inpatient treatment), augmented with community surveillance by CHWs to detect cases in a neighboring area. Findings suggest that CMAM delivered by CHWs is a cost-effective strategy compared with inpatient treatment, and compares well with the cost-effectiveness of other common child survival interventions.

The forest plots for this comparison are provided in the supplementary file (Figures S7 and S8).

Comparison 3: Facility-based strategies to screen and manage uncomplicated SAM according to the WHO protocol compared to other standards of care (inpatient treatment with RUTF compared to F100).

Three studies [57,59,62] assessed inpatient management of SAM with RUTF compared to F100. Among primary outcomes, there was no evidence of difference on weight gain (MD: 2 g/kg/day; 95% CI: −0.23 to 4.23; three studies; 266 participants; I^2^: 95%; very low quality outcome) and mortality (RR: 1.20; 95% CI: 0.34 to 4.22; two studies; 168 participants; I^2^: 16%; low quality outcome) in facility-based treatment with RUTF compared to F100. Among secondary outcomes, there was no difference between RUTF and F100 for height (MD: −0.59 mm/day; 95% CI: −3.91 to 2.73; one study; 120 participants; low quality outcome), MUAC (MD: −0.66 mm/day; 95% CI: −4.78 to 3.46; one study; 120 participants; low quality outcome), or wasting (RR: 1.47; 95% CI: 0.85 to 2.54; one study; 120 participants; low quality outcome).

The forest plots for this comparison are provided in the supplementary file (Figures S9 and S10).

Comparison 4: Community-based management of children with uncomplicated SAM as outpatients with RUTF compared to standard diet, fortified blended flours (FBFs), or other locally produced foods.

Fourteen studies [25,26,27,29,31,32,36,37,39,42,48,51,54,55] compared community-based management of children with uncomplicated SAM with RUTF versus other foods. Standard milk/peanut butter-based RUTF was compared with non-milk/peanut butter-based RUTF, reduced milk/peanut butter RUTF, F100, energy dense homemade food, and high oleic RUTF elevated n3 PUFA RUTF.

**Figure 3:**
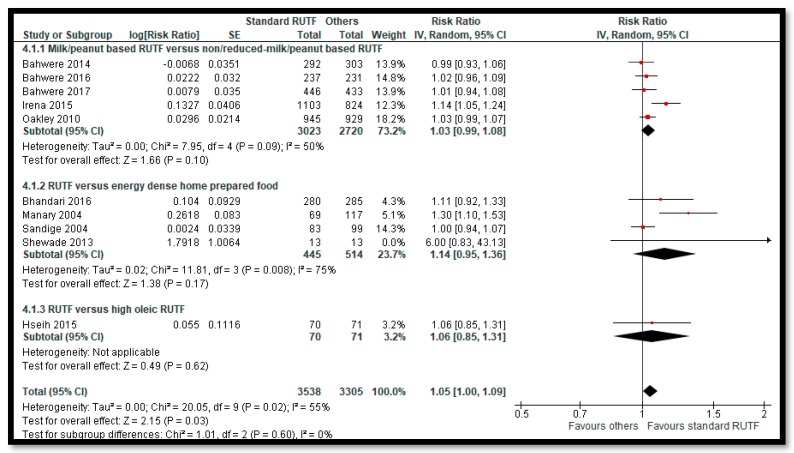
Impact of Community-based RUTF compared to other foods on recovery.

**Figure 4:**
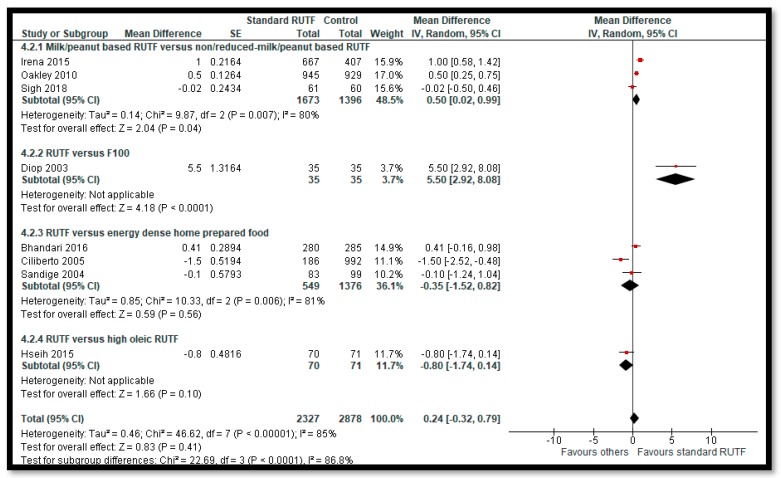
Impact of Community-based RUTF compared to other foods on Weight Gain (g/kg/day).

Among primary outcomes, there was no evidence of difference on recovery rate (Figure 3) when standard RUTF was compared to non-milk/peanut butter-based RUTF (RR: 1.03; 95% CI: 0.99 to 1.08; five studies; 5743 participants; I^2^ 50%; moderate quality outcome), energy-dense, home-prepared food (RR: 1.14; 95% CI 0.95 to 1.36; four studies; 959 participants; I^2^ 75%; low quality outcome), or high oleic RUTF (RR: 1.06; 95% CI: 0.85 to 1.31; one study; 141 participants; moderate quality outcome). Standard RUTF probably improves weight gain by 0.5 g/kg/day (Figure 4) when compared to non-milk/peanut butter-based RUTF (MD: 0.5 g/kg/day; 95% CI: 0.02 to 0.99; three studies; 3069 participants; I^2^ 80%; low quality outcome) and by 5.5 g/kg/day when compared to F100 (MD: 5.50 g/kg/day; 95% CI: 2.92 to 8.08; one study; 70 participants; low quality outcome). There was no evidence of difference on weight gain when standard RUTF was compared with energy-dense, home-prepared food (MD: −0.35 g/kg/day; 95% CI: −1.52 to 0.82; three studies; 1925 participants; I^2^ 81%; low quality outcome) and high oleic RUTF (MD: −0.8 g/kg/day; 95% CI: −1.74 to 0.14; one study; 141 participants; moderate quality outcome). There was no evidence of difference on mortality when standard RUTF was compared with non-milk/peanut butter-based RUTF (RR: 0.90; 95% CI: 0.72 to 1.12; five studies; 5743 participants; I^2^ 3%; moderate quality outcome), energy-dense, home-prepared food (RR: 1.87; 95% CI: 0.95 to 3.7; two studies; 1743 participants; I^2^ 0%; moderate quality outcome), high oleic RUTF (RR: 5.07; 95% CI: 0.61 to 42.31; one study; 141 participants; low quality outcome), and elevated n3 PUFA RUTF (RR: 0.33; 95% CI: 0.04 to 2.94; one study; 40 participants; low quality outcome) (Supplementary Figure S11).

Among secondary outcomes, there was no evidence of difference on height gain when standard RUTF was compared with non-milk/peanut butter-based RUTF (MD: −0.56 mm/day; 95% CI: −2.29 to 1.17; two studies; 1037 participants; I^2^ 63%; low quality outcome) and high oleic RUTF (MD: −0.09 mm/day; 95% CI: −0.21 to 0.03; one study; 141 participants; moderate quality outcome). Standard RUTF may improve height gain by 0.07 mm/day when compared to energy dense home food (−0.07 mm/day; 95% CI: −0.11 to −0.02; two studies; 1360 participants; I^2^ 0%; moderate quality outcome). There was no evidence of difference on MUAC gain when standard RUTF was compared with non-milk/peanut butter-based RUTF (MD: 0.68 mm/day; 95% CI: 0.00 to 1.36; three studies; 2111 participants; I^2^ 97%; low quality outcome), energy-dense, home-prepared food (MD: −0.03 mm/day; 95% CI: −0.15 to 0.08; two studies; 1360 participants; I^2^ 81%; low quality outcome), and high oleic RUTF (MD: −0.07 mm/day; 95% CI: −0.17 to 0.03; one study; 141 participants; moderate quality outcome). RUTF might reduce the time to recovery by 3.9 days when compared with F100 (MD: −3.9 days; 95% CI: −6.04 to −1.76; one study; 70 participants; low quality outcome) and by 1.2 days when compared with energy-dense, home-prepared food (MD: −1.21 days; 95% CI: −1.92 to −0.5; one study; 565 participants; low quality outcome). There was no difference between standard RUTF and other foods for any of adverse events, including coughing (RR: 0.97; 95% CI: 0.44 to 2.16; two studies; 1093 participants; I^2^ 84%; low quality outcome), diarrhea (RR: 1.01; 95% CI: 0.83 to 1.22; three studies; 1154 participants; I^2^ 0%; moderate quality outcome), and fever (RR: 1.21; 95% CI: 0.61 to 2.39; two studies; 1154 participants; I^2^ 88%; low quality outcome). There was no difference between standard RUTF and other foods for hospitalization (RR: 0.80; 95% CI: 0.46, 1.39; three studies; 2479 participants; I2 55%; low quality outcome).

The forest plots for this comparison are provided in the supplementary file (Figures S12–S16).

Comparison 5: RUSF for MAM compared to standard diet, FBF, or other locally produced foods.

Fourteen studies [23,34,40,41,43,45,46,47,49,53,56,58,61] compared RUSF for MAM with other foods. Other foods included whey RUSF, energy-dense, home-prepared food, CSB, and food supplements. Among primary outcomes, there was no evidence of difference on recovery rate when standard RUSF was compared to local or homemade food (RR: 0.92; 95% CI: 0.64 to 0.33; three studies; 435 participants; I^2^: 82%; low quality outcome), while RUSF probably reduces recovery rate when compared to whey RUSF by 4% (RR: 0.96; 95% CI: 0.92 to 1.00; one study; 2230 participants; high quality outcome). RUSF may improve recovery rate by 7% when compared to CSB (RR: 1.07; 95% CI: 1.02 to 1.13; six studies; 5744 participants; I^2^: 66%; low quality outcome). There was no evidence of difference on weight gain when RUSF was compared with local homemade food (MD: −0.75 g/kg/day; 95% CI: −2.03 to 0.43; one study; 73 participants; low quality outcome) and whey RUSF (MD: −0.16 g/kg/day; 95% CI: −0.33 to 0.01; one study; 2230 participants; high quality outcome). When compared to CSB, RUSF may improve weight gain. (MD: 0.49 g/kg/day; 95% CI: 0.10 to 0.87; five studies; 4354 participants; I^2^: 87%; low quality outcome). There was no evidence of difference on mortality when RUSF was compared to whey RUSF (RR: 2.11; 95% CI: 0.39 to 11.48; one study; 2230 participants; high quality outcome), CSB (RR: 0.92; 95% CI: 0.51 to 1.67; six studies; 5744 participants; moderate quality outcome), and food supplement (RR: 0.56; 95%CI: 0.05; 6.08; one study; 336 participants; low quality outcome).

Among secondary outcomes, there was no evidence of difference on height or length gain when RUSF was compared with local or homemade food (MD: −0.11; 95% CI: −0.50 to 0.28; three studies; 890 participants; I^2^: 72%; low quality outcome), whey RUSF (MD: −0.01; 95% CI: −0.03 to 0.01; one study; 2230 participants; high quality outcome), and CSB (MD: −0.00; 95% CI: −0.02 to 0.01; five studies; 4185 participants). RUSF may improve MUAC gain when compared with local or homemade food (MD: 0.22; 95% CI: 0.03 to 0.41; two studies; 817 participants; I^2^: 51%; low quality outcome), whey RUSF (MD: 0.04; 95% CI: 0.02 to 0.06; one study; 2230 participants; high quality outcome), and CSB (MD: 0.09; 95% CI: 0.04 to 0.13; seven studies; 5698 participants; I^2^: 53%; low quality outcome). RUSF may reduce time to recovery when compared to local or homemade food by 14 days (MD: −14.20 days; 95% CI: −26.08 to −2.32; one study; 55 participants; low quality outcome). There was no evidence of difference on time to recovery when RUSF was compared with whey RUSF (MD: −1.10 days; 95% CI: −2.73 to 0.53; one study; 2230 participants; high quality outcome) and CSB (MD: −2.77 days; 95% CI: −8.39 to 2.86; three studies; 3256 participants; I^2^: 99%; low quality outcome). There was no evidence of difference on moderate stunting when RUSF was compared with local or homemade food (MD: 0.85; 95% CI: 0.69 to 1.05; one study; 170 participants; low quality outcome). There was no evidence of difference on moderate wasting when RUSF was compared with whey RUSF (RR: 1.22; 95% CI: 0.34 to 4.39; one study; 170 participants; low quality outcome) and CSB (RR: 0.93; 0.69 to 1.27; one study; 1369 participants; low quality outcome). RUSF probably reduces severe wasting by 26% (RR: 0.74; 95% CI: 0.57 to 0.95; three studies; 3256 participants; I^2^: 0%; moderate quality outcome). There was no evidence of difference on underweight status when RUSF was compared with local or homemade food (RR: 1.06; 95% CI: 0.93 to 1.22; one study; 170 participants; low quality outcome). There was no difference between RUSF and other foods for fever (RR: 1.44; 95% CI: 0.95 to 2.18; one study; 2083 participants; moderate quality outcome), diarrhea (RR: 1.08; 95% CI: 0.96 to 1.22; three studies; 4022 participants; I^2^: 0%; moderate quality outcome), acute lower respiratory tract infection (ALRI) (RR: 0.98; 95% CI: 0.75 to 1.29; one study; 2083 participants; moderate quality outcome), other illnesses (RR: 0.78; 95% CI: 0.56 to 1.07; one study; 2083 participants; moderate quality outcome), any adverse events (RR: 1.17; 95% CI: 0.61 to 2.27; one study; 133 participants; low quality outcome), and severe adverse events (RR: 2.03; 95% CI: 0.53 to 7.78; one study; 133 participants; low quality outcome). RUSF may increase vomiting compared to other foods (RR: 1.39; 95% CI: 1.03 to 1.86; two studies; 1939 participants; low quality outcome). There was no difference in hospitalization between RUSF and other foods (RR: 0.76; 95% CI: 0.34, 1.70; five studies; 4140 participants; I^2^ 35%; low quality outcome).

The forest plots for this comparison are provided in the supplementary file (Figures S17–S28).

Comparison 6: Prophylactic use of antibiotics in children with uncomplicated SAM compared to no antibiotics.

**Figure 5:**
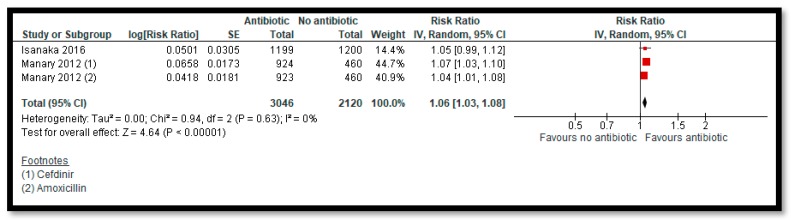
Impact of Prophylactic Antibiotic on Recovery Rate.

**Figure 6:**
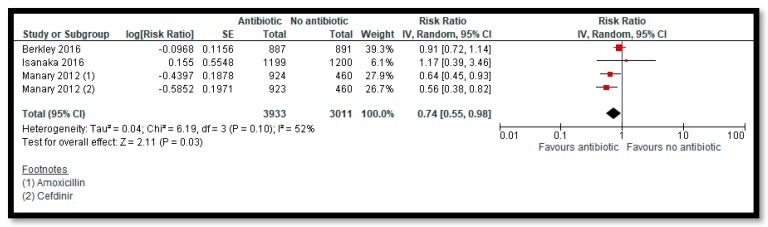
Impact of Prophylactic Antibiotics on Mortality.

Three studies [28,38,64] compared prophylactic use of antibiotics in children with uncomplicated SAM to no antibiotics. The antibiotics used for prophylaxis included co-trimoxazole [28], amoxicillin [38,64], and cefdinir [64]. Among the primary outcomes, antibiotics improve recovery by 6% (Figure 5) (RR: 1.06; 95% CI: 1.03, 1.08; two studies; 5166 participants; high quality outcome; I^2^ = 0%) and probably improves weight gain by 0.67 g/kg/day (MD: 0.67; 95% CI: 0.28, 1.06; two studies; 5052 participants; I^2^ = 66%; moderate quality outcome). Prophylactic antibiotic administration probably reduces mortality by 26% compared to the no antibiotics group (Figure 6) (RR: 0.74; 95% CI: 0.55, 0.98; three studies; 6944 participants; moderate quality outcome; I^2^ = 52%).

Among the secondary outcomes, prophylactic antibiotic administration probably improves MUAC by 0.06 mm/day compared to the control group (MD: 0.06 mm/day; 95% CI: 0.04, 0.08; two studies; 5031 participants; I^2^ = 0%; high quality outcome). There was no evidence of difference on length gain (MD: 0.01; 95% CI: −0.01, 0.04; two studies; 5052 participants; moderate quality outcome; I^2^ = 49%) or time to recovery (MD: −0.25; 95% CI: −1.55, 1.05; one study; 2442 participants; moderate quality outcome) when antibiotic was compared to no antibiotic. Three studies reported adverse events, suggesting no evidence of difference on diarrhea (RR: 0.96; 95% CI: 0.80, 1.16; three studies; 6707 participants; I^2^ = 88%; moderate quality outcome) or fever (RR: 0.95; 95% CI: 0.88, 1.04; two studies; 4926 participants; I^2^ = 0%; high quality outcome) between the antibiotic and no antibiotic groups. Prophylactic antibiotic probably decreases ARI symptoms compared to the no antibiotics by 11% (RR: 0.89; 95% CI: 0.83, 0.96; three studies; 6703 participants; high quality outcome; I^2^ = 36%). Prophylactic antibiotic administration reduces hospitalization by 11% compared to no antibiotic (RR: 0.89; 95% CI: 0.82, 0.98; three studies; 6944 participants; I^2^ = 0%; high quality outcome).

The other forest plots for this comparison are provided in the supplementary file (Figures S29–S34).

Comparison 7: Vitamin A supplementation in the management of SAM and MAM with various doses and frequency of administration.

Two studies [33,52] compared high dose vitamin A supplement with low dose vitamin A supplement. Among primary outcomes, there was no evidence of difference on weight gain at 2 weeks when high dose was compared to low dose vitamin A supplementation (MD: 0.05 g/kg/day; 95% CI: −0.08, 0.18; one study; 207 participants; moderate quality outcome). There was no evidence of difference on mortality at 15 days when high dose was compared to low dose vitamin A supplementation (RR: 7.07; 95% CI: 0.37, 135.13; one study; 207 participants; moderate quality outcome). Among the secondary outcomes, high dose vitamin A supplementation probably increases height gain by 0.1 cm compared to the low dose group (MD: 0.10; 95% CI: 0.02, 0.18; one study; 207 participants; moderate quality outcome). There was no evidence of difference on MUAC gain (MD: 0.80; 95% CI: −0.46, 2.06; one study; 207 participants; moderate quality outcome) or adverse events, including fever (RR: 1.50; 95% CI: 0.45, 5.05; one study; 122 participants; moderate quality outcome) and ALRI (RR: 1.00; 95% CI: 0.07, 13.87; one study; 20 participants; moderate quality outcome), when high dose vitamin A supplementation was compared with low dose supplementation.

The forest plots for this comparison are provided in the supplementary file (Figures S35−S39).

## 4. Discussion

This review summarizes findings from a total of 42 studies (from 48 papers), including 35,017 children. Thirty-three of the included studies were RCTs, six studies were quasi-experimental studies, and three studies were cost studies. All the studies were conducted in either community, hospital, health center, or nutrition rehabilitation center settings in LMICs, including Bangladesh, Mali, Malawi, Congo, Kenya, India, Niger, Senegal, Sudan, Burkina Faso, Zambia, Ethiopia, Sierra Leonne, Cameroon, Indonesia, and Cambodia.

Two studies assessed integrated community-based strategies to screen, identify, and manage MAM and SAM compared to non-community-based strategies. Integrated community-based management probably improves recovery rate by 4% and probably decreases weight gain by 0.8 g/kg/day compared to non-community-based management, while mortality was similar between the two group. Four studies assessed facility-based strategies to screen and manage uncomplicated SAM compared to other standards of care. Findings suggest that there was no evidence of difference on recovery or mortality. Three studies assessed facility-based management of SAM with RUTF compared to F100. There was no evidence of difference on weight gain or mortality when facility-based RUTF was compared with F100. Fourteen studies compared community-based management of children with uncomplicated SAM with RUTF versus other foods. There was no evidence of difference on recovery rate when standard RUTF was compared to other foods. Standard RUTF probably improves weight gain by 0.5 g/kg/day when compared to non-milk/peanut butter-based RUTF and by 5.5 g/kg/day when compared to F100, with no evidence of difference on weight gain when standard RUTF was compared with energy-dense, home-prepared food and high oleic RUTF. There was no evidence of difference on mortality when standard RUTF was compared with other foods. Fourteen studies compared RUSF for MAM with other foods. There was no evidence of difference on recovery when RUSF was compared to local or homemade food, while RUSF probably reduces recovery rate when compared to whey RUSF by 4%. RUSF probably improves recovery rate by 7% when compared to CSB. There was no evidence of difference on weight gain when RUSF was compared with local homemade food and whey RUSF, while RUSF may improve weight gain by 0.49 g/kg/day when compared with CSB. There was no evidence of difference on mortality when RUSF was compared with other foods. Three studies compared prophylactic use of antibiotics in children with uncomplicated SAM with no antibiotics. Prophylactic antibiotic therapy improves recovery by 6%, probably improves weight gain by 0.67 g/kg/day, and probably reduces mortality by 26% compared to no antibiotics. Two studies compared high dose vitamin A supplementation with low dose vitamin A supplementation in children with SAM. There was no evidence of difference on weight gain and mortality when high-dose was compared to low-dose vitamin A supplementation. The majority of the outcomes were rated as either moderate or low quality outcomes. Outcomes were downgraded mainly due to study limitations, high heterogeneity, imprecision, and small sample size.

To the best of our knowledge, this is the only comprehensive systematic review evaluating the interventions to manage acute malnutrition in children under five years of age in LMICs. Various systematic reviews have assessed the effectiveness of individual interventions for managing malnutrition in children. A previous systematic review [10] evaluated the effectiveness of interventions for SAM, including the WHO protocol for inpatient management and community-based management with ready-to-use-therapeutic food (RUTF), as well as interventions for MAM in children under five years in LMIC. This review included 14 studies and suggested that there are still gaps in the knowledge that need to be filled to estimate effectiveness of overall treatment approaches for SAM and MAM. One review [65] assessed outpatient care of children with nutritional edema compared to treatment in inpatient care or to treatment of marasmus in outpatient care, suggesting that edematous malnutrition could plausibly be treated effectively in outpatient service. However, the quality of evidence was low, and further good quality studies in various settings are required before conclusive guidance can be generated. Findings from our review suggests that the outpatient management probably improves recovery compared to the inpatient group, while there was no evidence of impact on mortality. Findings from the included studies on cost-effectiveness concluded that the cost for inpatient care and rehabilitation was significantly higher compared to daycare or ambulatory care services. Findings from our review provide a number of implications for future research, however further studies are needed to compare the effectiveness of various community and facility-based strategies, including active community-based surveillance, training of CHWs for community-based screening, and training of health facility staff to diagnose and treat uncomplicated SAM. Limited data also hindered the planned subgroup analysis based on age, duration of intervention, various formulations of supplementary foods, various settings, vitamin A dosage, various antibiotics, and equity. Future studies should be planned considering these research gaps. A recent Cochrane review [14] assessed the effects of home-based RUTF used during the rehabilitation phase of SAM in children on recovery, relapse, mortality, and rate of weight gain, suggesting that compared to alternative dietary approaches, standard RUTF probably improves recovery and may increase rate of weight gain slightly, but the effects on relapse and mortality are unknown. A review [12] assessed the efficacy and safety of home-based management of SAM using RUTF and compared it to F100 and home-based diet. Findings from this review suggested that the use of RUTF for home-based management of uncomplicated SAM was safe and efficacious, which is similar to the findings of our review. This findings are similar to the conclusions of our review. Our findings are in concordance with the results in [66], suggesting the current evidence supports the continued use of broad-spectrum oral amoxicillin for treating children with uncomplicated SAM as outpatients. Our findings also suggest beneficial effect of prophylactic antibiotic administration on recovery, weight gain, morality, and MUAC gain.

## 5. Conclusions

Findings from this review suggest that there are limited data comparing community-based management and facility-based management with other standard of care for SAM or MAM, suggesting some benefit of integrated community-based and outpatient management on improving recovery when compared to standard care and inpatient management. Existing cost data also suggest that community or outpatient management of children with uncomplicated SAM is the cost-effective strategy. Evidence also suggests that facility-based management of SAM with RUTF is similar to F100 on outcomes of weight gain and mortality. Existing evidence on RUTF suggests that standard RUTF is comparable with other foods for recovery and mortality for SAM; however, standard RUTF may improve weight gain when compared to non-milk/peanut butter-based RUTF and F100. Standard RUTF might also reduce recovery time when compared with F100 and energy-dense, home-prepared food. Existing data on RUSF suggest that RUSF may improve recovery and weight gain when compared with CSB for MAM. Data on prophylactic antibiotic administration in children with uncomplicated SAM suggest improved recovery rate and weight gain along with reduced mortality when compared to no antibiotic administration. Limited data suggest that high dose vitamin A supplementation is comparable with low dose vitamin A supplementation for weight gain and mortality among children with SAM.

Future studies are needed to evaluate the effectiveness of community and facility-based strategies for screening, identifying, and managing SAM and MAM, including studies comparing the effectiveness of various community and facility-based strategies, such as active community-based surveillance; training of CHWs for community-based screening; and training of health facility staff to diagnose and treat children with uncomplicated SAM. Existing data on the effectiveness of vitamin A supplementation are also limited, hence future data are needed to evaluate the role of vitamin A supplementation with various doses and frequency of administration among children with SAM and MAM. Future studies assessing the effectiveness of interventions to prevent and manage malnutrition among children in LMIC should report pertinent nutrition specific outcomes, including stunting, wasting, underweight status, infections, and potential adverse effects.

## Figures and Tables

**Table 1:** Characteristics of the Included Studies.

Study	Study Design	Setting	Participants	Intervention/Control	Outcomes
Comparison 1: Community-based strategies to screen, identify, and manage SAM and MAM compared to standard care					
Maust 2015	cRCT	Study carried out in 10 centers in Sierra Leone treating global acute malnutrition in children	1957 children aged 6–59 months	**Group 1: Integrated** (*N* = 1100) Children with SAM were given RUTF (175 kcal/kg/day) and amoxicillin every 2 weeks for 12 weeks. Children with MAM were given RUTF (75 kcal/kg/day) every 2 weeks for 12 weeks. **Group 2: Standard** (*N* = 857) Children with SAM were given RUTF (200 kcal/kg/day) and amoxicillin every 2 weeks for 12 weeks. Children with MAM were given super cereal plus (a fortified flour of CSB with oil and milk powder (1250 kcal/day)) every 2 weeks for 12 weeks	Coverage and recovery rate, duration of treatment, rates of weight and MUAC gain, clinical status. and cost of foodstuffs used
Wilford 2012	Cost-effectiveness study	District Dowa, Central Malawi	-	The study assessed the cost-effectiveness of community-based management of acute malnutrition (CMAM) to prevent deaths due to SAM	Costs and cost-effectiveness
Comparison 2: Facility-based strategies to screen and manage uncomplicated SAM according to the WHO protocol compared to other standards of care					
Ashworth 1994	RCT	The Children’s Nutrition Unit in central Dhaka, Bangladesh	573 children aged 12–60 months	**Group 1: Inpatient:** (*N* = 200) Children were admitted with their mothers and were resident until they reached 80% weight/height **Group 2: Daycare** (*N* = 200) Children came with their mothers from 0800 to 1700 every day except Friday, until 80% weight/height was reached **Group 3: Care at home** (*N* = 173) Children were treated in the daycare facility for 7 days (or up to 9 days if poor appetite or poor clinical outcome)	Cost-effectiveness, mortality, rate of edema loss, weight gain, and days taken to achieve 80% edema-free weight/height
Chapko 1994	RCT	Niger’s National Hospital, Niamey, Niger	100 malnourished children	**Group 1** (*N* = 53) Hospital-based rehabilitation **Group 2** (*N* = 47) Ambulatory-based rehabilitation	Cost of care, mortality, and anthropometric measures
Hossain 2008	Quasi-experimental	Urban setting in Dhaka, Bangladesh	60 children aged 2–59 months	**Group 1** (*N* = 30) Children were managed as per the WHO protocol **Group 2** (*N* = 30) Children were managed as per the Institute of Child and Mother Health (ICMH) protocol	Clinical determinants, improved appetite, disappearance of edema, improvement of other associated medical conditions. Catch-up growth
Puett 2013	Cost-effectiveness study	Rural setting in Bhola district, Bangladesh	-	The cost-effectiveness of community-based management for severe acute malnutrition (SAM) was compared with the “standard of care” for SAM (i.e., inpatient treatment), augmented with community surveillance by CHWs to detect cases in a neighboring area	Cost-effectiveness
Comparison 3: Facility-based strategies to screen and manage uncomplicated SAM according to the WHO protocol compared to other standards of care (inpatient treatment with RUTF compared to F100)					
Mishra 2018	RCT	Pediatrics ward of SCB Medical College, Cuttack, India	120 children aged 6–60 months	**Group 1: Locally prepared ready-to-use therapeutic food** (*N* = 60) **Group 2: F100** (*N* = 60)	Weight gain, recovery rate and length of stay, anthropometric determinants (weight, height, MUAC), clinical determinants (wasting, edema, death)
Thakur 2013	Quasi-experimental	Urban setting in Maharashtra, India	98 children ages 6–60 months	**Group 1: L-RUTF** (*N* = 50) Groundnut, milk powder, vegetable oil was given as 4 meals/day (12 g/kg/day), along with 4 meals from family pot **Group 2: F100-L** (*N* = 54) F100 locally produced was given as 60 mL/kg/day in 4 quarters + 4 meals from family food (total 120 kcal/kg/day).	Weight gain, duration of hospital stay
Versloot 2017	RCT	Blantyre, Malawi	74 children aged 6–60 months	**Group 1: RUTF-F75** (*N* = 26) Low protein milk-based formula diet given daily for 7 days (135 kcal/kg/day) **Group 2: F100** (*N* = 25) F100 milk diet given daily for 7 days (135 kcal/kg/day) **Group 3: RUTF** (*N* = 23) RUTF given daily for 7 days (135 kcal/kg/day)	Fecal pH, duration of stay, days with diarrhea, duration of edema, weight at discharge, hypo- and hypernatremia, reversion to F75 diet, and mortality
Comparison 4: Community-based management of children with uncomplicated SAM as outpatients with RUTF compared to standard diet, fortified blended flours (FBFs) or other locally produced foods					
Bahwere 2014	RCT	Lilong Health District, Central Malawi	600 children aged 6–59 months of age	**Group 1** (*N* = 308) Whey protein concentrate 34% (replacing dried skimmed milk (DSM)) was given weekly. One-week ratio *N* = 175 kcal/kg **Group 2** (*N* = 292) Peanut-based RUTF (P-RUTF) was given weekly	Average weight gain and recovery rate, length of stay (LOS)
Bahwere 2016	RCT	Study was carried out in a rural setting in Kabare administrative zone of South Kivu province, Democratic Republic of Congo	886 children; 6–23 months (*N* = 414), 24–59 months (*N* = 472)	**Group 1** (*N* = 445) Soya–maize–sorghum RUTF **Group 2** (*N* = 441) Standard peanut-paste-based RUTF	Recovery rate; mean daily weight gain; mean length of stay; hemoglobin change; differences in fat mass, body fat percentage, and fat mass index; fat-free mass and fat-free mass index; bio-electrical impedance analysis; illness marker and plasma concentrations of 8 key amino acids
Bahwere 2017	RCT	Study was carried out in 3 districts: Lilongwe, Dedza, Mchinji of Malawi; 21 clusters in each district	1347 children; *N* = 823 (6–23 months), *N* = 524 (24–59 months)	**Group 1: Peanut butter RUTF** (*N* = 454) Peanut butter, milk powder, sugar, vegetable oil, vit/min was given daily (0800 to 1600) until discharged or weight was gained **Group 2: Amino-acid enriched milk-free, soya, maize, sorghum** (*N* = 458) Amino-acid enriched milk-free soya, maize, sorghum was given daily (0800–1600 h) until discharged or weight was gained **Group 3: Amino acid enriched low cow milk** (*N* = 435) Amino acid enriched low-fat cow’s milk (9.3%), soya, maize, sorghum given daily (0800–1600 h) until discharged or weight was gained	Recovery rate, mean length of stay, mean daily weight gain, hemoglobin levels, body iron stores, RUTF intake, and morbidity
Bhandari 2016	RCT	Study was carried out in a mixed setting of Rajasthan, Delhi, and Tamil Nadu areas of India	906 children aged 6–59 months	**Group 1: RUTF (Commercial)** (*N* = 298) Commercial peanut paste, sugar, milk solids, vegetable oil, min/vit mix given weekly for 16 weeks **Group 2: RUTF (Local)** (*N* = 307) Local peanut paste, sugar, milk solids, vegetable oil, min/vit mix given weekly for 16 weeks **Control: Energy dense home prepared food** (*N* = 301) Cereals, pulses, sugar, oil, milk, eggs, min/vit mix given weekly for 16 weeks	Recovery weight gain, time to recovery, prevalence of diarrhea, acute lower respiratory tract infection (ALRI) and fever, mortality, and hospitalizations
Ciliberto 2005	Quasi-experimental	Study was carried out in a rural setting in South Malawi	1178 children aged 10–60 months	**Group 1** (*N* = 992) Home-based therapy with RUTF (HBT-RUTF) **Group 2** (*N* = 186) F100 standard inpatient therapy	Case fatality rate, successful recovery, relapse or death, rates of growth in body weight, MUAC, and length. Number of days of fever, cough, and diarrhea
Diop 2003	RCT	Urban setting in Rebuss, Dakar, Senegal	70 children aged 6–36 months	**Group 1: RUTF** (*N* = 35) Peanut-butter-based (Nutriset) given 3 times/day until discharged **Control: F100** (*N* = 35) Skim milk-based (Nutriset) given 3 times/day until discharged	Weight gain, food intake
Hseih 2015	RCT	Rural setting in Katana health district, South Kivu, Democratic Republic of Congo	141 children aged 6–59 months	**Group 1: High oleic RUTF:** (*N* = 71) High oleic peanuts, palm oil, and linseed oil given every 2 weeks for 12 weeks **Group 2: RUTF** (*N* = 70) Peanuts, palm oil, soy oil given every 2 week for 12 weeks	Change in plasma DHA and EPA content, rates of recovery length and weight gain, and change in plasma content of arachidonic acid
Irena 2015	cRCT	Health care clinics run by the Lusaka District Health Management Team in Lusaka, Zambia	1927 children aged 6–59 months	**Group 1: Standard RUTF** (*N* = 1103) Standard peanut-based RUTF given daily until discharged **Group 2:** Soybean, maize, and sorghum **RUTF** (*N* = 824) Soybean, maize, and sorghum grains given weekly until discharged	Recovery (cure), death, default, transfer out of the catchment area, and non-recovery
Jones 2015	RCT	Rural setting in Kilifi county, Kenya	60 children aged 6–50 months	**Group 1: Standard peanut-based RUTF** (*N* = 21) **Group 2: Flax seed oil-containing RUTF** (*N* = 20) Flax seed oil-based RUTF given weekly, except oil was given for 2 weeks only, followed by RUTF alone **Group 3: Flax seed oil-containing RUTF with additional fish oil capsules** (*N* = 20) Given weekly	Erythrocyte n-3 PUFA content, safety, and acceptability of the intervention; recovery and growth
Manary 2004	Quasi-experimental	Nutrition unit in Blantyre, Malawi	282 children aged 12–59 months	**Group 1: RTUF** plus supplement (*N* = 96) High energy diet + supplement **Group 2: Maize-Soy** (*N* = 117) **Group 3: RTUF** (*N* = 69) High Energy diet	Recovery rate, dropout, mortality, relapse, weight gain, height gain, MUAC gain
Oakley 2010	RCT	Rural setting in southern region of Malawi	1874 children aged 6–59 months	**Group 1: RUTF-10%** (*N* = 929) Skimmed milk (10%), whole soy flour (15%), peanut paste, min/vit mix was provided every 2 weeks for 8 weeks **Group 2: RUTF-25%** (*N* = 945) Skim milk (25%), peanut paste, min/vit mix was given weekly for 8 weeks	Recovery, rate of weight gain, and height gain
Sandige 2004	Quasi-experimental	Blantyre, Malawi	182 children aged 1–5 years	**Group 1: Local RUTF** (*N* = 99) Local RUTF composed of full fat milk powder, icing sugar, cotton seed oil, peanut butter and a mineral/vitamin mixture was given every 2 weeks for 16 weeks or until target weight was achieved **Group 2: Imported RUTF** (*N* = 83) Imported Plumpy’Nut (Nutriset) was given every 2 weeks for 16 weeks or until target weight was achieved	Recovery, weight gain, statural growth, growth in MUAC, anthropometric status, and the prevalence of fever, cough, and diarrhea
Shewade 2013	RCT	Urban setting in Chandigarh, India	26 children aged 6 months to 5 years	**Intervention group:** (*N* = 13) RUTF Groundnut-based diet prepared by program staff was provided on weekly basis for 12 weeks. Diet supplied 200 kcal/kg/d **Control group:** (*N* = 13) Supplementary nutrition from the anganwadi as per guidelines for management for malnutrition under the Integrated Child Development Scheme (ICDS)	Weight gain, WHZ, HAZ, WAZ, consumption
Sigh 2018	RCT	National Pediatric Hospital in Phnom Penh, Cambodia	121 children aged 6–59 months	**Group 1: NumTrey fish-based RUTF:** (*N* = 60) Two week rations of fish-based RUTF wafers (160 and 180 kcal/kg) based on weight were provided at each follow-up visit **Group 2: Milk based RUTF (BP100)** (*N* = 61) Two week rations of a standard product BP-100™ (160 and 180 kcal/kg)	Weight gain, height, MUAC, WHZ, WAZ, and HAZ
Comparison 5: RUSF for MAM compared to standard diet, FBF, or other locally produced foods					
Ackatia-Armah 2015	cRCT	Twelve community health centers in rural setting in Diola health district, Bamako, Mali	1264 children aged 6–35 months	**Group 1: Ready-to-use supplementary food (RUSF)** (*N* = 344) **Group 2: Corn soy blend (CSB++)** (*N* = 349) **Group 3: Misoloa (MI)** (*N* = 307) **Group 4: Locally milled flours + micronutrient powder (LMF)** (*N* = 284)	Adherence to treatment, MUAC, body weight and length, WHZ, HAZ, anemia, iron deficiency, iron deficiency anemia, hemoglobin, plasma ferritin, retinol binding protein, transferrin receptor, body iron stores, plasma zinc
Fabiansen 2017	RCT	Province de Passore, Burkina Faso	1609 children aged 6–23 months	**Group 1: LNS** (*N* = 809) Lipid-based nutrient supplement was given every 2 weeks for 12 weeks **Group 2: CSB** (*N* = 800) Corn/soy blend was given every 2 weeks for 12 weeks	Fat free mass index, recovery rate, anthropometric measures
Karakochul 2012	cRCT	10 health centers and health posts in the northern region of the Sidama zone, Ethiopia	1125 children aged 6–60 months	**Group 1: RUSF** (*N* = 375) Supplementary Plumpy’Nut (Nutriset) was given biweekly for 16 weeks **Group 2: CSB** (*N* = 750) CSB: Corn/soy blend + vegetable oil (premix) was given biweekly for 16 weeks	Recovery, default, transport, non-response, mortality
La Grone 2012	RCT	Rural setting in South TFC, Malawi	2890 children aged 6–59 months	**Group 1: CSB++** (*N* = 948) **Group 2: Soy RUSF** (*N* = 964) **Group 3: Soy/whey RUSF** (*N* = 978)	Recovered and developed SAM, remaining MAM, death, default time to recovery, rate of adverse events, and rates of gain in weight, length, and MUAC
Matilsky 2009	RCT	Rural setting in southern region of Malawi	1362 children aged 6–60 months	**Group 1:** **Milk/peanut fortified spread (Nutriset)** (*N* = 465) Given every 2 weeks for 8 weeks **Group 2:** **Soy/peanut fortified spread (Nutriset)** (*N* = 450) Given every 2 weeks for 8 weeks **Group 3:** **Corn-Soy Blend** (*N* = 447) Given every 2 weeks for 8 weeks	Recovery; rates of gain in weight, stature, and mid-upper arm circumference (MUAC); and adverse outcomes
Medoua 2015	RCT	Health districts of Mvog-Beti and Evodoula in the central region of Cameroon	81 children aged 6–59 months	**Group 1: CSB+** (*N* = 41) Improved corn/soy blend: corn, soya, sugar, min/vit + soy oil was given every 2 weeks for 16 weeks. Treatment diet provided 40 kcal/kg/d **Group 2: RUSF** (*N* = 40) Ready-to-use supplementary food: soya, corn flour, peanut paste, sugar, soy oil, min/vit was given every 2 weeks for 16 weeks. Control diet provided 40 kcal/kg/d	Recovery rate, time to recovery; and rates of gain in weight and mid-upper arm circumference
Nackers 2010	RCT	Two supplementary feeding centers (SFCs) in the remote and difficult-to-access villages of Mallawa and Bangaza (Magaria department, Zinder region, South of Niger)	807 children aged 6–59 months	**Group 1: CSB** (*N* = 406) Corn/soy blend premix + vegetable oil + sugar was given weekly for 16 weeks **Group 2: RUTF-Nutriset** (*N* = 401) (Plumpy’Nut) Peanut, powder milk, vegetable oil, vit/min mix was given weekly for 16 weeks	Weight gain and the recovery rate, mortality, non-responder and defaulter rates, length of stay, MUAC gain and hemoglobin gain, relapse and height gain
Nikiema 2014	cRCT	Rural setting in Hounde, Burkina Faso	1974 children aged 6–24 months of age	**Group 1: Child centered counselling** (*N* = 605) Only education counselling was given weekly for 12 weeks. No supplementation was provided **Group 2: Corn soy blend (CSB++)** (*N* = 675) Maize, soybean, milk, soy oil, vit/min mix diet was provided weekly for 12 weeks **Group 3: RUSF** (*N* = 694) Peanut butter, vegetable oil, whole soybean, shea butter, micronutrient-based diet was provided weekly for 12 weeks	Recovery, death, or drop-out; attendance; time to recovery; weight; length; daily MUAC gains
Phuka 2009	RCT	Rural setting in Lungwena, Mangochi District, Malawi	176 children aged 6–18 months	**Group 1: LP fortified** (*N* = 86) Maize flour, soya flour, micronutrient diet was given (71 g/d) weekly for 12 weeks **Group 2:** RUSF (*N* = 90) Maize flour-peanut butter, milk, vegetable oil, micronutrient diet was given (50 g/d) weekly for 12 weeks	Weight gain, length gain, mean change in anthropometric indices WAZ, LAZ, WLZ, recovery, change in MUAC, change in blood hemoglobin
Scherbaum 2015	Quasi-experimental	Nias Island, Indonesia	129 children under five years of age	**Group 1: Peanut/milk-based spreads program** (*N* = 44) Peanut/milk-based spread was given for 4 to 6 weeks or until recovered **Group 2: CNL-B: Cereal/nut/legume-based biscuits program** (*N* = 47) Cereal/nut/legume-based biscuits were given for 4 to 6 weeks or until recovered **Group 3: CNL-B and intensive nutrition education (INE)** (*N* = 38) Cereal/nut/legume-based biscuits + intensive nutrition education were given for 4 to 6 weeks or until recovered	Weight, height, WHZ, recovery, compliance
Singh 2010	RCT	Rural setting in Vellore, India	118 children aged 18–60 months	**Group 1: RUTF** (*N* = 61) **Group 2: High caloric cereal meal** (*N* = 57) High calorie cereal milk (HCCM) supplement	Recovery; changes in vitamin B12, plasma Zinc, serum albumin levels, and iron status
Stobough 2016	RCT	Rural setting in South Malawi/Mozambique border residents	2259 children aged 6–59 months of age	**Group 1: Whey protein RUSF:** (*N* = 1144) A dairy-based, whey protein, whey permeate concentrate (75 kcal/kg/day) was given every 2 weeks for 12 weeks **Group 2: soy-flour RUSF** (*N* = 2086) Extruded soy flour (75 kcal/kg/day) was given every 2 weeks for 12 weeks	Recovery; changes in MUAC, weight, and length; time to recovery; any adverse events
Thakwalakwa 2010	RCT	Rural setting of Lungwena, Mangochi district of Malawi	189 children aged 6–15 months	**Group 1: CSB** (*N* = 67) Corn/soy blend given weekly for 12 weeks **Group 2: LNS** (*N* = 66) Peanut paste, dry skim milk, vegetable oil, sugar, min/vit mix given weekly for 12 weeks **Group 3: Control** (*N* = 59) Infants breastfed only	Weight change, length change, hemoglobin, WLZ, LAZ, MUAC, head circumference, adverse events
Vanelli 2014	RCT	Makeni, Northern region, Sierra Leonne	332 children aged 6–60 months	**Group 1: Feeding Program supplementations** (*N* = 177) **Group 2** (*N* = 159) 100 g servings of “Parma pap” equal to the weekly requirement containing peanut, palm oil, milk, mineral/vitamin mix given weekly for 12 weeks	Weight, length, WHZ
Comparison 6: Prophylactic use of antibiotics in children with uncomplicated SAM compared to no antibiotics					
Berkley 2016	RCT	Study was carried out in four hospitals in Kenya (two rural hospitals in Kilifi and Malindi, and two urban hospitals in Mombasa and Nairobi)	1781 children aged 60 days to 59 months	**Group 1** (*N* = 887) Daily treatment with water dispersible co-trimoxazole tablets for 6 months **Group 2** (*N* = 891) Placebo given daily for 6 months	Mortality, frequency of non-fatal illness episodes resulting in readmission to hospital outpatient attendance; the clinical syndromes associated with death or illness; pathogens detected from blood culture, urine culture, and malaria testing; suspected toxic effects during the period that investigational products were received; and changes in MUAC, weight-for-height, weight-for-length, weight-for-age, height-for-age, length-for-age, head circumference-for-age, and hematological indices
Isanaka 2016	RCT	Rural setting in Madarounfa, Niger	2412 children aged 6–59 months	**Intervention** (*N* = 1210) Twice daily treatment with a split-dose of 80 mg/kg of body weight with amoxicillin. Duration of treatment was 1 week. **Control** (*N* = 1202) Placebo administered two times per day for 1 week	Nutritional recovery by 8 weeks, non-response at 8 weeks, death from any cause, default, and transfer to inpatient care
Manary 2012	RCT	18 feeding clinics in rural Malawi	2767 children aged 6–59 months	**Group 1: Amoxicillin** (*N* = 924) Daily treatment with amoxicillin suspension of 80–90 mg/kg for initial 7 days of the therapy **Group 2: Cefdinir** (*N* = 923) Daily treatment with 14 mg/kg Cefdinir suspension for initial 7 days of the therapy **Group 3: Placebo** (*N* = 920) Placebo administered daily for initial 7 days of the therapy	Recovery rate, mortality, weight gain, length gain, antibiotics rates of adverse events, and time to recovery
Comparison 7: Vitamin A supplementation in the management of SAM and MAM with various doses and frequency of administration					
Donnen 1998	RCT	Rural setting in Katana health district, South Kivu, Democratic Republic of Congo	900 hospitalized preschool children aged 0–72 months	**Group 1** (*N* = 300) High dose Vitamin A (200,000 IU or 100, 000 IU (age < 12 months)) on day of admission followed by placebo for every subsequent day until discharge **Group 2** (*N* = 298) Low dose Vitamin A (5000 IU) on day of admission followed by placebo for every subsequent day until discharge **Control** (*N* = 302) Placebo administered until discharge	Morbidity, mortality, duration of hospitalization
Sattar 2012	RCT	Urban/peri-urban setting in Dhaka, Bangladesh	260 children aged 6–59 months	**Group 1: High dose Vitamin A** (*N* = 130) 200,000 IU or 100, 000 IU if aged < 12 months on day of admission followed by low dose (5000 IU) on each subsequent day for 15 days **Group 2: Placebo** (*N* = 130) Administered on day of admission followed by low dose vitamin A (5000 IU) each day for 15 days	Clinical success, adverse events; clinical features of vitamin A toxicity, changes in serum retinol and RBP levels, duration of resolution of diarrhea, ALRI, edema, dermatosis, other illness, changes in weight and length or height, nosocomial morbidities and mortality

SAM: Sever acute malnutrition; MAM: Moderate acute malnutrition; cRCT: Cluster randomized trials; RUTF: Ready-to-use therapeutic food; MUAC: Mid-upper arm circumferences; CHW: Community health workers; F75: Formula 75; F100: Formula 100; WHZ: Weight-for-height Z-score; HAZ: Height-for-age Z-score; WAZ: Weight-for-age Z-score; RUSF: Ready-to-use supplementary food; RBP: Retinol binding protein; ALRI: Acute lower respiratory infections; LNS: Lipid based nutrient supplement; CSB: Corn soy blend.

## Supplementary Materials

The following are available online at https://www.mdpi.com/2072-6643/12/1/116/s1, Figure S1: Forest plot for the impact of community based strategies compared to no community based strategies on Recovery. Figure S2: Forest plot for the impact of community based strategies compared to no community based strategies on Weight Gain. Figure S3: Forest plot for the impact of community based strategies compared to no community based strategies on Mortality. Figure S4: Forest plot for the impact of community based strategies compared to no community based strategies on Length Gain. Figure S5: Forest plot for the impact of community based strategies compared to no community based strategies on MUAC Gain. Figure S6: Forest plot for the impact of community based strategies compared to no community based strategies on Adverse Events. Figure S7: Forest plot for the impact of facility based strategies according to WHO protocol compared to other protocols on Recovery. Figure S8: Forest plot for the impact of facility based strategies according to WHO protocol compared to other protocols on Mortality. Figure S9: Forest plot for the impact of facility based treatment with RUTF compared to F100 on Weight Gain. Figure S10: Forest plot for the impact of facility based treatment with RUTF compared to F100 on Mortality. Figure S11: Forest plot for the impact of RUTF compared to other foods on Mortality. Figure S12: Forest plot for the impact of RUTF compared to other foods on Height/Length Gain. Figure S13: Forest plot for the impact of RUTF compared to other foods on MUAC Gain. Figure S14: Forest plot for the impact of RUTF compared to other foods on Time to Recovery. Figure S15: Forest plot for the impact of RUTF compared to other foods on Adverse Events. Figure S16: Forest plot for the impact of RUTF compared to other foods on Hospitalisation. Figure S17: Forest plot for the impact of RUSF for MAM compared to other foods on Recovery. Figure S18: Forest plot for the impact of RUSF for MAM compared to other foods on Weight Gain. Figure S19: Forest plot for the impact of RUSF for MAM compared to other foods on Mortality. Figure S20: Forest plot for the impact of RUSF for MAM compared to other foods on Length/Height Gain. Figure S21: Forest plot for the impact of RUSF for MAM compared to other foods on MUAC. Figure S22: Forest plot for the impact of RUSF for MAM compared to other foods on Time to Recovery. Figure S23: Forest plot for the impact of RUSF for MAM compared to other foods on Moderate Stunting. Figure S24: Forest plot for the impact of RUSF for MAM compared to other foods on Moderate Wasting. Figure S25: Forest plot for the impact of RUSF for MAM compared to other foods on Severe Wasting. Figure S26: Forest plot for the impact of RUSF for MAM compared to other foods on Moderate Underweight. Figure S27: Forest plot for the impact of RUSF for MAM compared to other foods on Adverse Events. Figure S28: Forest plot for the impact of RUSF for MAM compared to other foods on Hospitalisation. Figure S29: Forest plot for the impact of prophylactic antibiotic compared to no antibiotic on Weight Gain. Figure S30: Forest plot for the impact of prophylactic antibiotic compared to no antibiotic on MUAC Gain. Figure S31: Forest plot for the impact of prophylactic antibiotic compared to no antibiotic on Length Gain. Figure S32: Forest plot for the impact of prophylactic antibiotic compared to no antibiotic on Time to Recovery. Figure S33: Forest plot for the impact of prophylactic antibiotic compared to no antibiotic on Adverse Events. Figure S34: Forest plot for the impact of prophylactic antibiotic compared to no antibiotic on Hospitalisation. Figure S35: Forest plot for the impact of vitamin A supplementation on Weight Change. Figure S36: Forest plot for the impact of vitamin A supplementation on Mortality. Figure S37: Forest plot for the impact of vitamin A supplementation on Height Change. Figure S38: Forest plot for the impact of vitamin A supplementation on MUAC Change. Figure S39: Forest plot for the impact of vitamin A supplementation on Adverse Events.

## Appendix A. Search Strategy

PubMed Search Strategy (searched in title, abstract and/or keyword searches)

#1. “Infant” [Mesh]

#2. “Child, Preschool” [Mesh]

#3. Infant*

#4. Toddler*

#5. Baby OR babies

#6. Newborn* OR Neonat*

#7. Preschool* OR Kindergarten* OR Under-5s OR “Under 5s” OR “Under 5”

#8. #1 OR #2 OR #3 OR #4 OR #5 OR #6 OR #7

#9. “Severe Acute Malnutrition” [Mesh]

#10. “Infant Nutrition Disorders” [Mesh]

#11. “Nutrition Disorders” [Mesh]

#12. “Severe Acute Malnutrition” OR SAM

#13. “Moderate Acute Malnutrition” OR MAM

#14. “Protein-Energy Malnutrition” [Mesh]

#15. Undernutrition OR under-nutrition

#16. Malnourish*

#17. Malnutrition

#18. Stunted OR wasted OR wasting OR “Wasting Syndrome” [Mesh]

#19. Starve* OR Starvat* OR “Starvation” [Mesh]

#20. “Vitamin A” OR “Vitamin A Deficiency” “Vitamin A” [Mesh]

#21. “Iron” [Mesh] OR “Iron deficiency” OR “Fe deficiency” OR “Anemia” [Mesh]

#22. Zinc OR “Zinc deficiency OR “Zn deficiency” OR “Zinc” [Mesh]

#23. #9 OR #10 OR #11 OR #12 OR #13 OR #14 OR #15 OR #16 OR #17 OR #18 OR #19 OR #20 OR #21 OR #22

#24. “Food” [Mesh]

#25. “Infant Food” [Mesh]

#26. “Food, Fortified” [Mesh]

#27. “Food, Formulated” [Mesh]

#28. “Dietary Supplements” [Mesh]

#29. “Fortified Food*”

#30. “Diet* Supplement*”

#31. “Ready to use therapeutic food” OR RUTF

#32. “Ready to use supplementary food” OR RUSF

#33. “Ready to use food*” OR RUF

#34. F100 OR F75

#35. CTC

#36. “Vitamin A Supplement*”

#37. “Micronutrient* Supplement*”

#38. “Dietary Fats” [Mesh]

#39. “Dietary Proteins” [Mesh]

#40. FBF

#41. “Corn soy*”

#42. “Wheat soy* blend*”

#43. “Rice mild blend*”

#44. “Milk rice blend*”

#45. “Pea wheat blend*”

#46. “Cereal pulse blend*”

#47. “Lipid-based nutrient supplement*”

#48. Nutributter

#49. “Milk Proteins” [Mesh]

#50. “Community-based management of malnutrition” OR CMAM

#51. “Amoxicillin” [Mesh]

#52. “Cotrimoxazole” [Mesh]

#53. Bacteraemia*

#54. Gentamicin

#55. “Penicillin G” [Mesh]

#56. “Chloramphenicol” [Mesh]

#57. “Ceftriaxone” [Mesh]

#58. “Ciprofloxacin” [Mesh]

#59. “Inpatient management” OR “In-patient management” OR IMCI OR IMNCI

#60. “Community-based management”

#61. “Facility-based management”

#62. Prophyla* AND antibiotic*

#63. #24 OR #25 OR #26 OR #27 OR #28 OR #29 OR #30 OR #31 OR #32 OR #33 OR #34 OR #35 OR #36 OR #37 OR #38 OR #39 OR #40 OR #41 OR #42 OR #43 OR #44 OR #45 OR #46 OR #47 OR #48 OR #49 OR #50 OR #51 OR #52 OR #53 OR #54 OR #55 OR #56 OR #57 OR #58 OR #59 OR #60 OR #61 OR #62

#64. “Morbidity” [Mesh]

#65. “Mortality” [Mesh]

#66. Death*

#67. Relapse*

#68. Recovery

#69. #64 OR #65 OR #66 OR #67 OR #68

#70. #8 AND #23 AND (#63 OR #69)

#71. Age Filters Applied: Infants 1-23 months; birth-23 months; Preschool child 2–5 years

## Appendix B. Reasons for Exclusion for Excluded Studies

**Study**	**Reason for Exclusion**
Agha 2004 [67]	This study did not have an appropriate control group.
Aguayo 2018 [68]	The study design was not appropriate.
Ahmed 1999 [69]	The study design was not appropriate.
Ashworth 2004 [70]	The study design was not appropriate.
Bachou 2008 [71]	The study design was not appropriate.
Badaloo 1999 [72]	This study did not assess the intervention of interest; study compared high protein formula with low protein formula.
Baker 1978 [73]	The study did not assess the intervention of interest; study compared milk diet with soy-maize-porridge diet.
Bhandari 2001 [74]	The study did not assess the intervention of interest; study compared food supplementation with counselling with nutritional counselling alone.
Burza 2016 [75]	The study design was not appropriate.
Donnen 2007 [76]	This study included children up to 14 years of age
Dubray 2008 [77]	This study compared two different antibiotics (ceftriaxone vs amoxicillin) in children with SAM and did not have an appropriate control group (no antibiotic/placebo).
Javan 2017 [78]	This study was conducted in Upper Middle Income Country.
Linneman 2007 [79]	This study did not have an appropriate control group.
Nagar 2016 [80]	This study did not have an appropriate control group.
Roy 2005 [81]	The study did not assess the intervention of interest; study compared supplementary feeding with education to feeding alone.
Simpore 2006 [82]	This study did not have an appropriate control group.
Zongo 2013 [83]	The study did not assess the intervention of interest; the study compared Moringa leaf in addition to the usual porridge diet.

## References

[1] World Health Organization (WHO) (2017). Malnutrition. https://www.who.int/news-room/fact-sheets/detail/malnutrition.

[2] UNICEF (2009). Types of Undernutrition: Growthz Failure-UNICEF. https://www.unicef.org/nutrition/training/2.3/contents.html.

[3] Hawkes C. (2017). Global Nutrition Report 2017: Nourishing the SDGs. https://data.unicef.org/resources/global-nutrition-report-2017-nourishing-sdgs/.

[4] UNICEF, WHO (2019). International Bank for Reconstruction and Development/The World Bank, Levels and Trends in Child Malnutrition: Key Findings of the 2019 Edition of the Joint Child Malnutrition Estimates.

[5] Black R.E., Victora C.G., Walker S.P., Bhutta Z.A., Christian P., de Onis M., Ezzati M., Grantham-McGregor S., Katz J., Martorell R. (2013). Maternal and child undernutrition and overweight in low-income and middle-income countries. Lancet.

[6] Bhutta Z.A., Das J.K., Rizvi A., Gaffey M.F., Walker N., Horton S., Webb P., Lartey A., Black R.E., The Lancet Nutrition Interventions Review Group (2013). Evidence-based interventions for improvement of maternal and child nutrition: What can be done and at what cost?. Lancet.

[7] Ruel M.T., Alderman H., Maternal and Child Nutrition Study Group (2013). Nutrition-sensitive interventions and programmes: How can they help to accelerate progress in improving maternal and child nutrition?. Lancet.

[8] World Health Organization (WHO) (2013). Guideline: Updates on the Management of Severe Acute Malnutrition in Infants and Children.

[9] Picot J., Hartwell D., Harris P., Mendes D., Clegg A.J., Takeda A. (2012). The Effectiveness of Interventions to Treat Severe Acute Malnutrition in Young Children: A Systematic Review.

[10] Lenters L.M., Wazny K., Webb P., Ahmed T., Bhutta Z.A. (2013). Treatment of Severe and Moderate Acute Malnutrition in Low-and Middle-Income Settings: A Systematic Review, Meta-Analysis and Delphi Process. BMC Public Health.

[11] Alcoba G., Kerac M., Breysse S., Salpeteur C., Galetto-Lacour A., Briend A., Gervaix A. (2013). Do children with uncomplicated severe acute malnutrition need antibiotics? A systematic review and meta-analysis. PLoS ONE.

[12] Gera T. (2010). Efficacy and safety of therapeutic nutrition products for home based therapeutic nutrition for severe acute malnutrition: A systematic review. Indian Pediatr..

[13] Lazzerini M., Tickell D. (2011). Antibiotics in severely malnourished children: Systematic review of efficacy, safety and pharmacokinetics. Bull. World Health Organ..

[14] Schoonees A., Lombard M.J., Musekiwa A., Nel E., Volmink J. (2019). Ready-to-use therapeutic food (RUTF) for home-based nutritional rehabilitation of severe acute malnutrition in children from six months to five years of age. Cochrane Database Syst. Rev..

[15] Kristjansson E., Francis D.K., Liberato S., Benkhalti Jandu M., Welch V., Batal M., Greenhalgh T., Rader T., Noonan E., Shea B. (2015). Food supplementation for improving the physical and psychosocial health of socio-economically disadvantaged children aged three months to five years. Cochrane Database Syst. Rev..

[16] Visser J., McLachlan M.H., Fergusson P., Volmink J., Garner P. (2013). Supplementary feeding for food insecure, vulnerable and malnourished populations-an overview of systematic reviews. Cochrane Database Syst. Rev..

[17] Manary M., Iannotti L., Trehan I. (2012). Systematic Review of Vitamin A Supplementation in the Treatment of Children with Severe Acute Malnutrition.

[18] (2014). Review Manager.

[19] Higgins J.P., Green S. (2011). Cochrane Handbook for Systematic Reviews of Interventions.

[20] EPOC Resources for Review Authors 2017. Epoc.cochrane.org/resources/epoc-resources-review-authors.

[21] Guyatt G.H., Oxman A.D., Vist G.E., Kunz R., Falck-Ytter Y., Alonso-Coello P., Schunemann H.J., Group G.W. (2008). GRADE: An emerging consensus on rating quality of evidence and strength of recommendations. BMJ.

[22] Balshem H., Helfand M., Schunemann H.J., Oxman A.D., Kunz R., Brozek J., Vist G.E., Falck-Ytter Y., Meerpohl J., Norris S. (2011). GRADE guidelines: 3. Rating the quality of evidence. J. Clin. Epidemiol..

[23] Ackatia-Armah R.S., McDonald C.M., Doumbia S., Erhardt J.G., Hamer D.H., Brown K.H. (2015). Malian children with moderate acute malnutrition who are treated with lipid-based dietary supplements have greater weight gains and recovery rates than those treated with locally produced cereal-legume products: A community-based, cluster-randomized trial. Am. J. Clin. Nutr..

[24] Ashworth A., Huttly S., Khanum S. (1994). Controlled trial of three approaches to the treatment of severe malnutrition. Lancet.

[25] Bahwere P., Akomo P., Mwale M., Murakami H., Banda C., Kathumba S., Banda C., Jere S., Sadler K., Collins S. (2017). Soya, maize, and sorghum–based ready-to-use therapeutic food with amino acid is as efficacious as the standard milk and peanut paste–based formulation for the treatment of severe acute malnutrition in children: A noninferiority individually randomized controlled efficacy clinical trial in Malawi. Am. J. Clin. Nutr..

[26] Bahwere P., Balaluka B., Wells J.C.K., Mbiribindi C.N., Sadler K., Akomo P., Dramaix-Wilmet M., Collins S. (2016). Cereals and pulse-based ready-to-use therapeutic food as an alternative to the standard milk-and peanut paste–based formulation for treating severe acute malnutrition: A noninferiority, individually randomized controlled efficacy clinical trial. Am. J. Clin. Nutr..

[27] Bahwere P., Banda T., Sadler K., Nyirenda G., Owino V., Shaba B., Dibari F., Collins S. (2014). Effectiveness of milk whey protein-based ready-to-use therapeutic food in treatment of severe acute malnutrition in M alawian under-5 children: A randomised, double-blind, controlled non-inferiority clinical trial. Matern. Child Nutr..

[28] Berkley J.A., Ngari M., Thitiri J., Mwalekwa L., Timbwa M., Hamid F., Ali R., Shangala J., Mturi N., Jones K.D. (2016). Daily co-trimoxazole prophylaxis to prevent mortality in children with complicated severe acute malnutrition: A multicentre, double-blind, randomised placebo-controlled trial. Lancet Glob. Health.

[29] Bhandari N., Mohan S.B., Bose A., Iyengar S.D., Taneja S., Mazumder S., Pricilla R.A., Iyengar K., Sachdev H.S., Mohan V.R. (2016). Efficacy of three feeding regimens for home-based management of children with uncomplicated severe acute malnutrition: A randomised trial in India. BMJ Glob. Health.

[30] Chapko M.K., Prual A., Gamatie Y., Maazou A.A. (1994). Randomized clinical trial comparing hospital to ambulatory rehabilitation of malnourished children in Niger. J. Trop. Pediatr..

[31] Ciliberto M.A., Sandige H., Ndekha M.J., Ashorn P., Briend A., Ciliberto H.M., Manary M.J. (2005). Comparison of home-based therapy with ready-to-use therapeutic food with standard therapy in the treatment of malnourished Malawian children: A controlled, clinical effectiveness trial. Am. J. Clin. Nutr..

[32] Diop E.H.I., Dossou N.I., Ndour M.M., Briend A., Wade S. (2003). Comparison of the efficacy of a solid ready-to-use food and a liquid, milk-based diet for the rehabilitation of severely malnourished children: A randomized trial. Am. J. Clin. Nutr..

[33] Donnen P., Dramaix M., Brasseur D., Bitwe R., Vertongen F., Hennart P. (1998). Randomized placebo-controlled clinical trial of the effect of a single high dose or daily low doses of vitamin A on the morbidity of hospitalized, malnourished children. Am. J. Clin. Nutr..

[34] Fabiansen C., Yameogo C.W., Iuel-Brockdorf A.S., Cichon B., Rytter M.J.H., Kurpad A., Wells J.C., Ritz C., Ashorn P., Filteau S. (2017). Effectiveness of food supplements in increasing fat-free tissue accretion in children with moderate acute malnutrition: A randomised 2 × 2 × 3 factorial trial in Burkina Faso. PLoS Med..

[35] Hossain M.M., Hassan M.Q., Rahman M.H., Kabir A.R., Hannan A.H., Rahman A.K. (2009). Hospital management of severely malnourished children: Comparison of locally adapted protocol with WHO protocol. Indian Pediatr..

[36] Hsieh J.-C., Liu L., Zeilani M., Ickes S., Trehan I., Maleta K., Craig C., Thakwalakwa C., Singh L., Brenna J.T. (2015). High oleic ready-to-use therapeutic food maintains docosahexaenoic acid status in severe malnutrition: A randomized, blinded trial. J. Pediatr. Gastroenterol. Nutr..

[37] Irena A.H., Bahwere P., Owino V.O., Diop E.I., Bachmann M.O., Mbwili-Muleya C., Dibari F., Sadler K., Collins S. (2015). Comparison of the effectiveness of a milk-free soy-maize-sorghum-based ready-to-use therapeutic food to standard ready-to-use therapeutic food with 25% milk in nutrition management of severely acutely malnourished Z ambian children: An equivalence non-blinded cluster randomised controlled trial. Matern. Child Nutr..

[38] Isanaka S., Langendorf C., Berthe F., Gnegne S., Li N., Ousmane N., Harouna S., Hassane H., Schaefer M., Adehossi E. (2016). Routine amoxicillin for uncomplicated severe acute malnutrition in children. N. Engl. J. Med..

[39] Jones K.D.J., Ali R., Khasira M.A., Odera D., West A.L., Koster G., Akomo P., Talbert A.W.A., Goss V.M., Ngari M. (2015). Ready-to-use therapeutic food with elevated n-3 polyunsaturated fatty acid content, with or without fish oil, to treat severe acute malnutrition: A randomized controlled trial. BMC Med..

[40] Karakochuk C., van den Briel T., Stephens D., Zlotkin S. (2012). Treatment of moderate acute malnutrition with ready-to-use supplementary food results in higher overall recovery rates compared with a corn-soya blend in children in southern Ethiopia: An operations research trial. Am. J. Clin. Nutr..

[41] LaGrone L.N., Trehan I., Meuli G.J., Wang R.J., Thakwalakwa C., Maleta K., Manary M.J. (2011). A novel fortified blended flour, corn-soy blend ‘plus-plus,’is not inferior to lipid-based ready-to-use supplementary foods for the treatment of moderate acute malnutrition in Malawian children. Am. J. Clin. Nutr..

[42] Manary M.J., Ndkeha M.J., Ashorn P., Maleta K., Briend A. (2004). Home based therapy for severe malnutrition with ready-to-use food. Arch. Dis. Child..

[43] Matilsky D.K., Maleta K., Castleman T., Manary M.J. (2009). Supplementary feeding with fortified spreads results in higher recovery rates than with a corn/soy blend in moderately wasted children. J. Nutr..

[44] Maust A., Koroma A.S., Abla C., Molokwu N., Ryan K.N., Singh L., Manary M.J. (2015). Severe and moderate acute malnutrition can be successfully managed with an integrated protocol in Sierra Leone. J. Nutr..

[45] Medoua G.N., Ntsama P.M., Ndzana A.C., Essa’a V.J., Tsafack J.J., Dimodi H.T. (2016). Recovery rate of children with moderate acute malnutrition treated with ready-to-use supplementary food (RUSF) or improved corn–soya blend (CSB+): A randomized controlled trial. Public Health Nutr..

[46] Nackers F., Broillet F., Oumarou D., Djibo A., Gaboulaud V., Guerin P.J., Rusch B., Grais R.F., Captier V. (2010). Effectiveness of ready-to-use therapeutic food compared to a corn/soy-blend-based pre-mix for the treatment of childhood moderate acute malnutrition in Niger. J. Trop. Pediatr..

[47] Nikiema L., Huybregts L., Kolsteren P., Lanou H., Tiendrebeogo S., Bouckaert K., Kouanda S., Sondo B., Roberfroid D. (2014). Treating moderate acute malnutrition in first-line health services: An effectiveness cluster-randomized trial in Burkina Faso. Am. J. Clin. Nutr..

[48] Oakley E., Reinking J., Sandige H., Trehan I., Kennedy G., Maleta K., Manary M. (2010). A ready-to-use therapeutic food containing 10% milk is less effective than one with 25% milk in the treatment of severely malnourished children. J. Nutr..

[49] Phuka J., Thakwalakwa C., Maleta K., Cheung Y.B., Briend A., Manary M., Ashorn P. (2009). Supplementary feeding with fortified spread among moderately underweight 6–18-month-old rural Malawian children. Matern. Child Nutr..

[50] Puett C., Sadler K., Alderman H., Coates J., Fiedler J.L., Myatt M. (2012). Cost-effectiveness of the community-based management of severe acute malnutrition by community health workers in southern Bangladesh. Health Policy Plan..

[51] Sandige H., Ndekha M.J., Briend A., Ashorn P., Manary M.J. (2004). Home-based treatment of malnourished Malawian children with locally produced or imported ready-to-use food. J. Pediatr. Gastroenterol. Nutr..

[52] Sattar S., Ahmed T., Rasul C.H., Saha D., Salam M.A., Hossain M.I. (2012). Efficacy of a high-dose in addition to daily low-dose vitamin A in children suffering from severe acute malnutrition with other illnesses. PLoS ONE.

[53] Scherbaum V., Purwestri R.C., Stuetz W., Inayati D.A., Suryantan J., Bloem M.A., Biesalski H.K. (2015). Locally produced cereal/nut/legume-based biscuits versus peanut/milk-based spread for treatment of moderately to mildly wasted children in daily programmes on Nias Island, Indonesia: An issue of acceptance and compliance?. Asia Pac. J. Clin. Nutr..

[54] Shewade H.D., Patro B.K., Bharti B., Soundappan K., Kaur A., Taneja N. (2013). Effectiveness of indigenous ready-to-use therapeutic food in community-based management of uncomplicated severe acute malnutrition: A randomized controlled trial from India. J. Trop. Pediatr..

[55] Sigh S., Roos N., Chamnan C., Laillou A., Prak S., Wieringa F.T. (2018). Effectiveness of a locally produced, fish-based food product on weight gain among Cambodian children in the treatment of acute malnutrition: A randomized controlled trial. Nutrients.

[56] Singh A.S., Kang G., Ramachandran A., Sarkar R., Peter P., Bose A. (2010). Locally made ready-to-use therapeutic food for treatment of malnutrition: A randomized controlled trial. Indian Pediatr..

[57] Sm M., Rai P., Swain A.S.B. (2019). Locally prepared ready to use therapeutic food for the treatment of children with severe acute malnutrition: A randomised controlled trial. World J. Pharm. Med. Res..

[58] Stobaugh H.C., Ryan K.N., Kennedy J.A., Grise J.B., Crocker A.H., Thakwalakwa C., Litkowski P.E., Maleta K.M., Manary M.J., Trehan I. (2016). Including whey protein and whey permeate in ready-to-use supplementary food improves recovery rates in children with moderate acute malnutrition: A randomized, double-blind clinical trial. Am. J. Clin. Nutr..

[59] Thakur G.S., Singh H., Patel C. (2013). Locally-prepared ready-to-use therapeutic food for children with severe acute malnutrition: A controlled trial. Indian Pediatr..

[60] Thakwalakwa C., Ashorn P., Phuka J., Cheung Y.B., Briend A., Puumalainen T., Maleta K. (2010). A lipid-based nutrient supplement but not corn-soy blend modestly increases weight gain among 6-to 18-month-old moderately underweight children in rural Malawi. J. Nutr..

[61] Vanelli M., Virdis R., Contini S., Corradi M., Cremonini G., Marchesi M., Mele A., Monti F., Pagano B., Proietti I. (2014). A hand-made supplementary food for malnourished children. Acta Bio Med. Atenei Parm..

[62] Versloot C.J., Voskuijl W., van Vliet S.J., van den Heuvel M., Carter J.C., Phiri A., Kerac M., Heikens G.T., van Rheenen P.F., Bandsma R.H.J. (2017). Effectiveness of three commonly used transition phase diets in the inpatient management of children with severe acute malnutrition: A pilot randomized controlled trial in Malawi. BMC Pediatr..

[63] Wilford R., Golden K., Walker D.G. (2011). Cost-effectiveness of community-based management of acute malnutrition in Malawi. Health Policy Plan..

[64] Trehan I., Goldbach H.S., LaGrone L.N., Meuli G.J., Wang R.J., Maleta K.M., Manary M.J. (2016). Research Article (New England Journal of Medicine) Antibiotics as part of the management of severe acute malnutrition. Malawi Med. J..

[65] Roberfroid D., Hammami N., Mehta P., Lachat C., Verstraeten R., Weise Prinzo Z., Huybregts L., Kolsteren P. (2013). Management of Oedematous Malnutrition in Infants and Children Aged> 6 Months: A Systematic Review of the Evidence.

[66] Williams P.C., Berkley J.A. (2018). Guidelines for the treatment of severe acute malnutrition: A systematic review of the evidence for antimicrobial therapy. Paediatr. Int. Child Health.

[67] Agha S. (2004). Supplementary Feeding of Malnourished Children in Northern Iraq. East Mediterr Health J..

[68] Aguayo V.M., Badgaiyan N., Qadir S.S., Bugti A.N., Alam M.M., Nishtar N., Galvin M. (2018). Community management of acute malnutrition (CMAM) programme in Pakistan effectively treats children with uncomplicated severe wasting. Matern. Child Nutr..

[69] Ahmed T., Ali M., Choudhary I.A., Haque M.E., Salam M.A., Rabbani G.H., Suskind R.M., Fuchs G.J. (1999). Mortality in severely malnourished children with diarrhoea and use of a standardised management protocol. Lancet.

[70] Ashworth A., Chopra M., McCoy D., Sanders D., Jackson D., Karaolis N., Sogaula N., Schofield C. (2004). WHO guidelines for management of severe malnutrition in rural South African hospitals: Effect on case fatality and the influence of operational factors. Lancet.

[71] Bachou H., Tumwine J.K., Mwadime R.K., Ahmed T., Tylleskar T. (2008). Reduction of unnecessary transfusion and intravenous fluids in severely malnourished children is not enough to reduce mortality. Ann. Trop. Paediatr..

[72] Badaloo A., Boyne M., Reid M., Persaud C., Forrester T., Millward D.J., Jackson A.A. (1999). Dietary protein, growth and urea kinetics in severely malnourished children and during recovery. J. Nutr..

[73] Baker R., Baker S., Margo G., Reuter H. (1978). Successful use of a soya-maize mixture in the treatment of kwashiorkor. S. Afr. Med. J..

[74] Bhandari N., Bahl R., Nayyar B., Khokhar P., Rohde J.E., Bhan M.K. (2001). Food supplementation with encouragement to feed it to infants from 4 to 12 months of age has a small impact on weight gain. J. Nutr..

[75] Burza S., Mahajan R., Marino E., Sunyoto T., Shandilya C., Tabrez M., Kumar K., Jha A., Mathew P., Salse N. (2016). Seasonal effect and long-term nutritional status following exit from a community-based management of severe acute malnutrition program in Bihar, India. Eur. J. Clin. Nutr..

[76] Donnen P., Sylla A., Dramaix M., Sall G., Kuakuvi N., Hennart P. (2007). Effect of daily low dose of vitamin A compared with single high dose on morbidity and mortality of hospitalized mainly malnourished children in senegal: A randomized controlled clinical trial. Eur. J. Clin. Nutr..

[77] Dubray C., Ibrahim S.A., Abdelmutalib M., Guerin P.J., Dantoine F., Belanger F., Legros D., Pinoges L., Brown V. (2008). Treatment of severe malnutrition with 2-day intramuscular ceftriaxone vs 5-day amoxicillin. Ann. Trop. Paediatr..

[78] avan R., Kooshki A., Afzalaghaee M., Aldaghi M., Yousefi M. (2017). Effectiveness of supplementary blended flour based on chickpea and cereals for the treatment of infants with moderate acute malnutrition in Iran: A randomized clinical trial. Electron. Phys..

[79] Linneman Z., Matilsky D., Ndekha M., Manary M.J., Maleta K., Manary M.J. (2007). A large-scale operational study of home-based therapy with ready-to-use therapeutic food in childhood malnutrition in Malawi. Matern. Child Nutr..

[80] Nagar R.P., Nagar T., Gupta B.D. (2016). Treatment outcome in patients with severe acute malnutrition managed with protocolised care at malnutrition treatment corner in Rajasthan, India: A prospective observational study (quasi-experimental). Int. J. Res. Med. Sci..

[81] Roy S.K., Fuchs G.J., Mahmud Z., Ara G., Islam S., Shafique S., Akter S.S., Chakraborty B. (2005). Intensive nutrition education with or without supplementary feeding improves the nutritional status of moderately-malnourished children in Bangladesh. J. Health Popul. Nutr..

[82] Simpore J., Kabore F., Zongo F., Dansou D., Bere A., Pignatelli S., Biondi D.M., Ruberto G., Musumeci S. (2006). Nutrition rehabilitation of undernourished children utilizing Spiruline and Misola. Nutr. J..

[83] Zongo U., Zoungrana S.L., Savadogo A., Traoré A.S. (2013). Nutritional and clinical rehabilitation of severely malnourished children with Moringa oleifera Lam. leaf powder in Ouagadougou (Burkina Faso). Food Nutr. Sci..

