# Supplementary material for: Effectiveness of Interventions for Managing Acute Malnutrition in Children under Five Years of Age in Low-Income and Middle-Income Countries: A Systematic Review and Meta-Analysis
Source: Nutrients. 2020 Jan 1;12(1):116. doi: 10.3390/nu12010116 (PMC7019612; doi:10.3390/nu12010116)
Supplement: Supplementary file 1 [file nutrients-12-00116-s001.pdf]

Appendix A: Search Strategy

PubMed Search Strategy (searched in title, abstract and/or keyword searches)

#1. "Infant"[Mesh]

#2. "Child, Preschool"[Mesh]

#3. Infant*

#4. Toddler*

#5. Baby OR babies

#6. Newborn* OR Neonat*

#7. Preschool* OR Kindergarten* OR Under-5s OR "Under 5s" OR "Under 5"

#8. #1 OR #2 OR #3 OR #4 OR #5 OR #6 OR #7

#9. "Severe Acute Malnutrition"[Mesh]

#10. "Infant Nutrition Disorders"[Mesh]

#11. "Nutrition Disorders"[Mesh]

#12. "Severe Acute Malnutrition" OR SAM

#13. "Moderate Acute Malnutrition" OR MAM

#14. "Protein-Energy Malnutrition"[Mesh]

#15. Undernutrition OR under-nutrition

#16. Malnourish*

#17. Malnutrition

#18. Stunted OR wasted OR wasting OR "Wasting Syndrome"[Mesh]

#19. Starve* OR Starvat* OR "Starvation"[Mesh]

#20. "Vitamin A" OR "Vitamin A Deficiency" "Vitamin A"[Mesh]

#21. "Iron"[Mesh] OR "Iron deficiency" OR "Fe deficiency" OR "Anemia"[Mesh]

#22. Zinc OR "Zinc deficiency OR "Zn deficiency" OR "Zinc"[Mesh]

#23. #9 OR #10 OR #11 OR #12 OR #13 OR #14 OR #15 OR #16 OR #17 OR #18 OR #19 OR #20 OR #21 OR #22

#24. "Food"[Mesh]

#25. "Infant Food"[Mesh]

#26. "Food, Fortified"[Mesh]

#27. "Food, Formulated"[Mesh]

#28. "Dietary Supplements"[Mesh]

#29. "Fortified Food*"

#30. "Diet* Supplement*"

#31. "Ready to use therapeutic food" OR RUTF

#32. "Ready to use supplementary food" OR RUSF

#33. "Ready to use food*" OR RUF

#34. F100 OR F75

#35. CTC

#36. "Vitamin A Supplement*"

#37. "Micronutrient* Supplement*"

#38. "Dietary Fats"[Mesh]

#39. "Dietary Proteins"[Mesh]

#40. FBF

#41. "Corn soy*"

#42. "Wheat soy* blend*"

#43. "Rice mild blend*"

#44. "Milk rice blend*"

#45. "Pea wheat blend*"

#46. "Cereal pulse blend*"

#47. "Lipid-based nutrient supplement*"

#48. Nutributter

#49. "Milk Proteins"[Mesh]

#50. "Community based management of malnutrition" OR CMAM

#51. "Amoxicillin"[Mesh]

#52. "Cotrimoxazole"[Mesh]

#53. Bacteraemia*

#54. Gentamicin

#55. "Penicillin G"[Mesh]

#56. "Chloramphenicol"[Mesh]

#57. "Ceftriaxone"[Mesh]

#58. "Ciprofloxacin"[Mesh]

#59. "Inpatient management" OR "In-patient management" OR IMCI OR IMNCI

#60. "Community based management"

#61. "Facility based management"

#62. Prophyla* AND antibiotic*

#63. #24 OR #25 OR #26 OR #27 OR #28 OR #29 OR #30 OR #31 OR #32 OR #33 OR #34 OR #35 OR #36 OR #37 OR #38 OR #39 OR #40 OR #41 OR #42 OR #43 OR #44 OR #45 OR #46 OR #47 OR #48 OR #49 OR #50 OR #51 OR #52 OR #53 OR #54 OR #55 OR #56 OR #57 OR #58 OR #59 OR #60 OR #61 OR #62

#64. "Morbidity"[Mesh]

#65. "Mortality"[Mesh]

#66. Death*

#67. Relapse*

#68. Recovery

#69. #64 OR #65 OR #66 OR #67 OR #68

#70. #8 AND #23 AND (#63 OR #69)

#71. Age Filters Applied: Infants 1-23 months; birth-23 months; Preschool child 2-5 years

Appendix B: Reasons for exclusion for excluded studies

Study	Reason for Exclusion
Agha 2004 [1]	This study did not have an appropriate control group.
Aguayo 2018 [2]	The study design was not appropriate.
Ahmed 1999 [3]	The study design was not appropriate.
Ashworth 2004 [4]	The study design was not appropriate.
Bachou 2008 [5]	The study design was not appropriate.
Badaloo 1999 [6]	This study did not assess the intervention of interest; study compared high protein formula with low protein formula.
Baker 1978 [7]	The study did not assess the intervention of interest; study compared milk diet with soy-maize-porridge diet.
Bhandari 2001 [8]	The study did not assess the intervention of interest; study compared food supplementation with counselling with nutritional counselling alone.
Burza 2016 [9]	The study design was not appropriate.
Donnen 2007 [10]	This study included children up to 14 years of age
Dubray 2008 [11]	This study compared two different antibiotics (ceftriaxone vs amoxicillin) in children with SAM and did not have an appropriate control group (no antibiotic/placebo).
Javan 2017 [12]	This study was conducted in Upper Middle Income Country.
Linneman 2007 [13]	This study did not have an appropriate control group.
Nagar 2016 [14]	This study did not have an appropriate control group.
Roy 2005 [15]	The study did not assess the intervention of interest; study compared supplementary feeding with education to feeding alone.
Simpore 2006 [16]	This study did not have an appropriate control group.
Zongo 2013 [17]	The study did not assess the intervention of interest; the study compared Moringa leaf in addition to the usual porridge diet.

© 2019 by the authors. Submitted for possible open access publication under the terms and conditions of the Creative Commons Attribution (CC BY) license (http://creativecommons.org/licenses/by/4.0/).

Appendix C: Supplementary Figures

Comparison 1: Community based strategies to screen, identify and manage SAM and MAM compared to no community based strategies

Figure 1: Forest plot for the impact of community based strategies compared to no community based strategies on Recovery

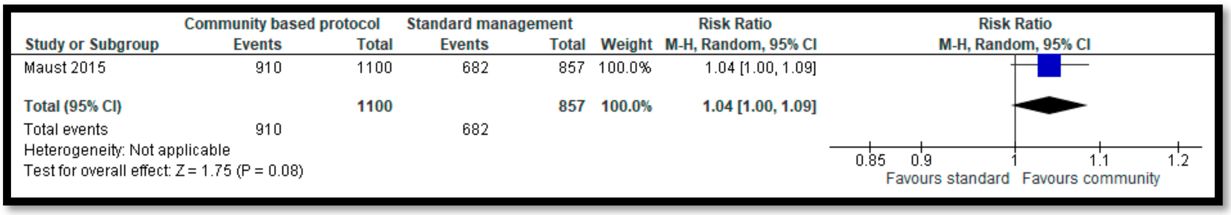

Figure 2: Forest plot for the impact of community based strategies compared to no community based strategies on Weight Gain

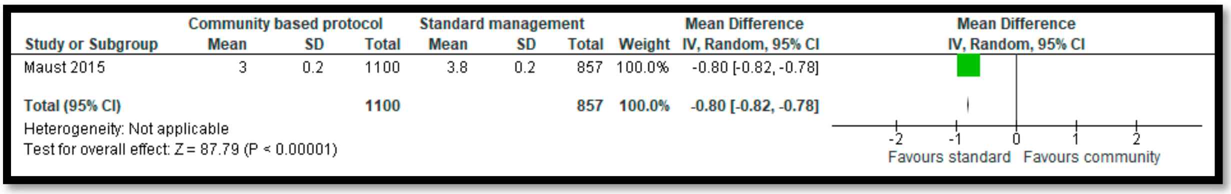

Figure 3: Forest plot for the impact of community based strategies compared to no community based strategies on Mortality

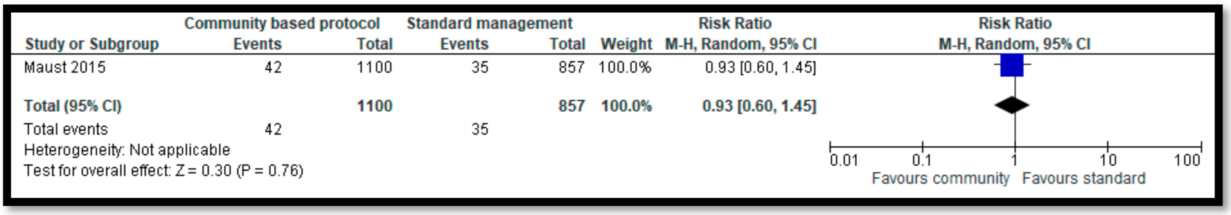

Figure 4: Forest plot for the impact of community based strategies compared to no community based strategies on Length Gain

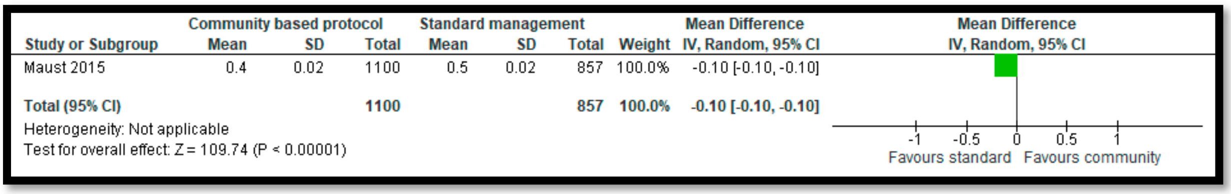

Figure 5: Forest plot for the impact of community based strategies compared to no community based strategies on MUAC Gain

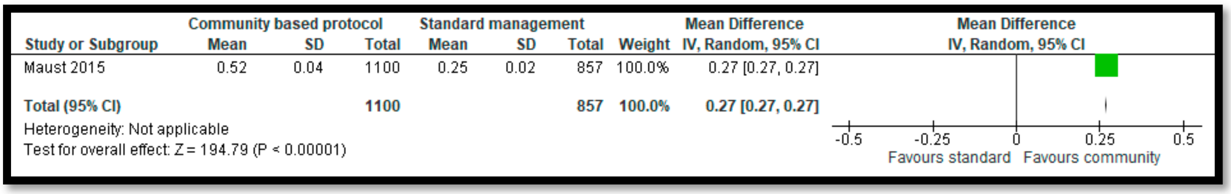

Figure 6: Forest plot for the impact of community based strategies compared to no community based strategies on Adverse Events

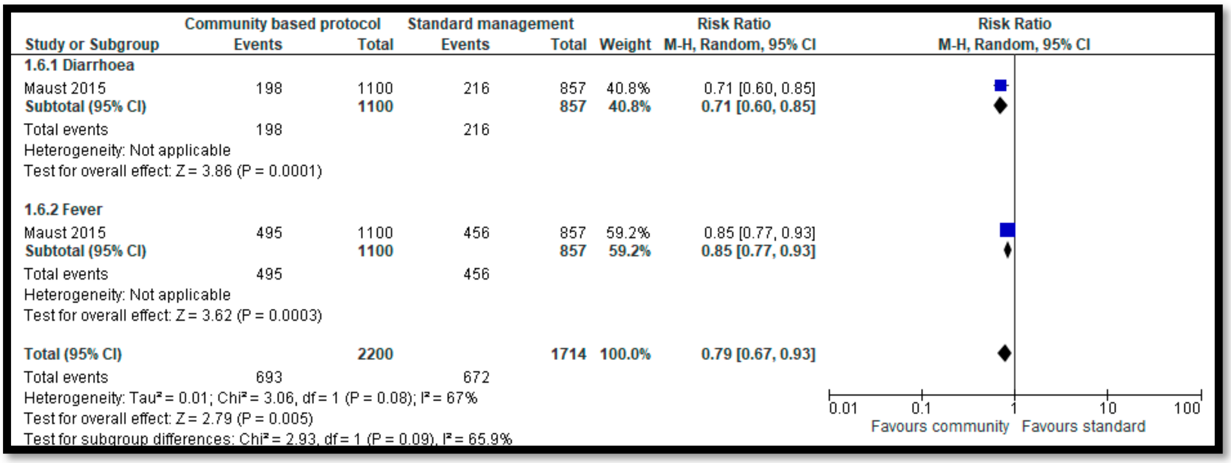

Study or Subgroup	Community based protocol Events	Community based protocol Total	Standard management Events	Standard management Total	Weight	Risk Ratio M-H, Random, 95% CI
**1.6.1 Diarrhoea**						
Maust 2015	198	1100	216	857	40.8%	0.71 [0.60, 0.85]
**Subtotal (95% CI)**		**1100**		**857**	**40.8%**	**0.71 [0.60, 0.85]**
Total events	198		216			
Heterogeneity: Not applicable						
Test for overall effect: Z = 3.86 (P = 0.0001)						
**1.6.2 Fever**						
Maust 2015	495	1100	456	857	59.2%	0.85 [0.77, 0.93]
**Subtotal (95% CI)**		**1100**		**857**	**59.2%**	**0.85 [0.77, 0.93]**
Total events	495		456			
Heterogeneity: Not applicable						
Test for overall effect: Z = 3.62 (P = 0.0003)						
**Total (95% CI)**		**2200**		**1714**	**100.0%**	**0.79 [0.67, 0.93]**
Total events	693		672			
Heterogeneity: Tau² = 0.01; Chi² = 3.06, df = 1 (P = 0.08); I² = 67%						
Test for overall effect: Z = 2.79 (P = 0.005)						
Test for subgroup differences: Chi² = 2.93, df = 1 (P = 0.09), I² = 65.9%						

Comparison 2: Facility based strategies to screen and manage uncomplicated SAM according to the WHO protocol compared to other standards of care

Figure 7: Forest plot for the impact of facility based strategies according to WHO protocol compared to other protocols on Recovery

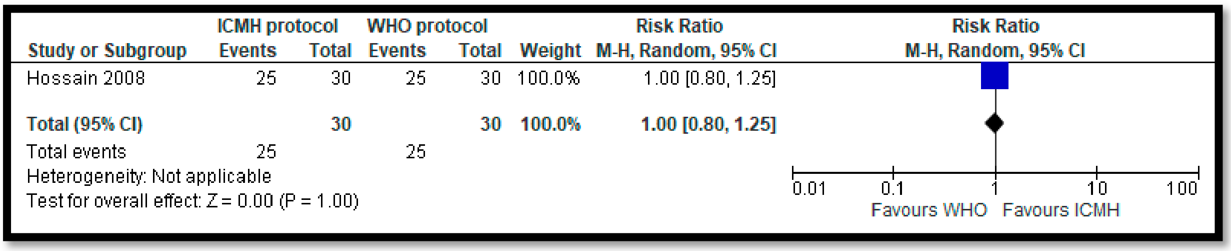

Study or Subgroup	ICMH protocol Events	ICMH protocol Total	WHO protocol Events	WHO protocol Total	Weight	Risk Ratio M-H, Random, 95% CI
Hossain 2008	25	30	25	30	100.0%	1.00 [0.80, 1.25]
**Total (95% CI)**		**30**		**30**	**100.0%**	**1.00 [0.80, 1.25]**
Total events	25		25			
Heterogeneity: Not applicable						
Test for overall effect: Z = 0.00 (P = 1.00)						

Figure 8: Forest plot for the impact of facility based strategies according to WHO protocol compared to other protocols on Mortality

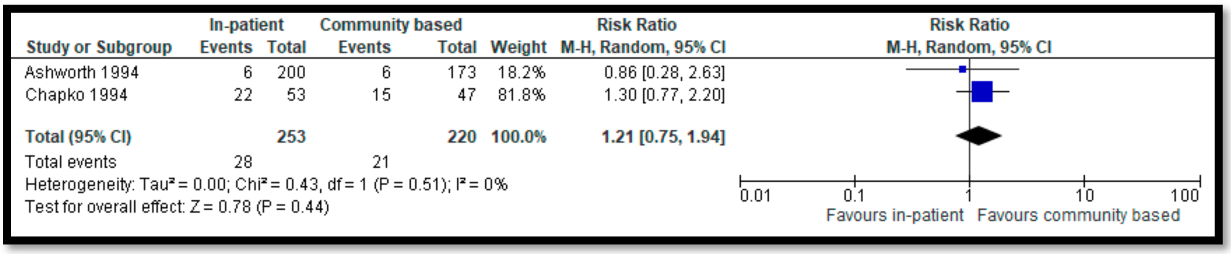

Study or Subgroup	In-patient Events	In-patient Total	Community based Events	Community based Total	Weight	Risk Ratio M-H, Random, 95% CI
Ashworth 1994	6	200	6	173	18.2%	0.86 [0.28, 2.63]
Chapko 1994	22	53	15	47	81.8%	1.30 [0.77, 2.20]
**Total (95% CI)**		**253**		**220**	**100.0%**	**1.21 [0.75, 1.94]**
Total events	28		21			
Heterogeneity: Tau² = 0.00; Chi² = 0.43, df = 1 (P = 0.51); I² = 0%						
Test for overall effect: Z = 0.78 (P = 0.44)						

Comparison 3: Facility based strategies to screen and manage uncomplicated SAM according to the WHO protocol compared to other standards of care (In-patient treatment with RUTF compared to F100)

Figure 9: Forest plot for the impact of facility based treatment with RUTF compared to F100 on Weight Gain

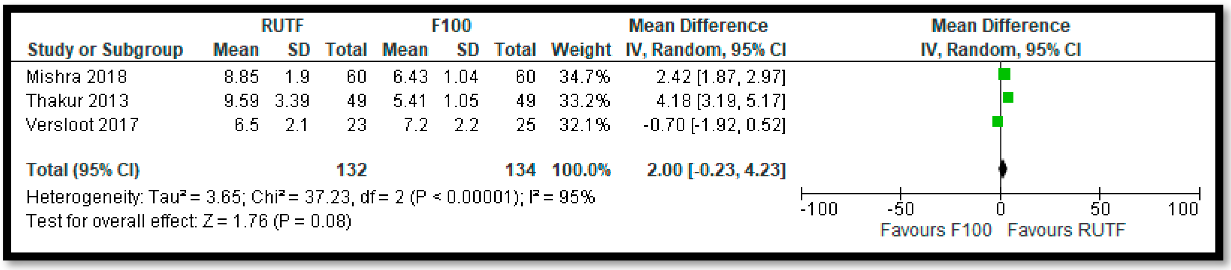

Study or Subgroup	RUTF Mean	RUTF SD	RUTF Total	F100 Mean	F100 SD	F100 Total	Weight	Mean Difference IV, Random, 95% CI
Mishra 2018	8.85	1.9	60	6.43	1.04	60	34.7%	2.42 [1.87, 2.97]
Thakur 2013	9.59	3.39	49	5.41	1.05	49	33.2%	4.18 [3.19, 5.17]
Versloot 2017	6.5	2.1	23	7.2	2.2	25	32.1%	-0.70 [-1.92, 0.52]
**Total (95% CI)**			**132**			**134**	**100.0%**	**2.00 [-0.23, 4.23]**

Heterogeneity: Tau² = 3.65; Chi² = 37.23, df = 2 (P < 0.00001); I² = 95%
Test for overall effect: Z = 1.76 (P = 0.08)

Figure 10: Forest plot for the impact of facility based treatment with RUTF compared to F100 on Mortality

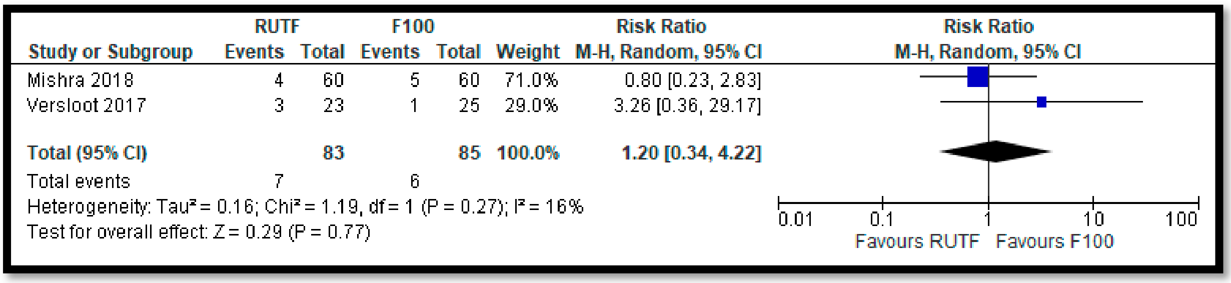

Study or Subgroup	RUTF Events	RUTF Total	F100 Events	F100 Total	Weight	Risk Ratio M-H, Random, 95% CI
Mishra 2018	4	60	5	60	71.0%	0.80 [0.23, 2.83]
Versloot 2017	3	23	1	25	29.0%	3.26 [0.36, 29.17]
**Total (95% CI)**		**83**		**85**	**100.0%**	**1.20 [0.34, 4.22]**
Total events	7		6			

Heterogeneity: Tau² = 0.16; Chi² = 1.19, df = 1 (P = 0.27); I² = 16%
Test for overall effect: Z = 0.29 (P = 0.77)

Comparison 4: Community based management of children with uncomplicated SAM as outpatients with RUTF compared to standard diet, fortified blended flours (FBFs) or other locally produced foods

Figure 11: Forest plot for the impact of RUTF compared to other foods on Mortality

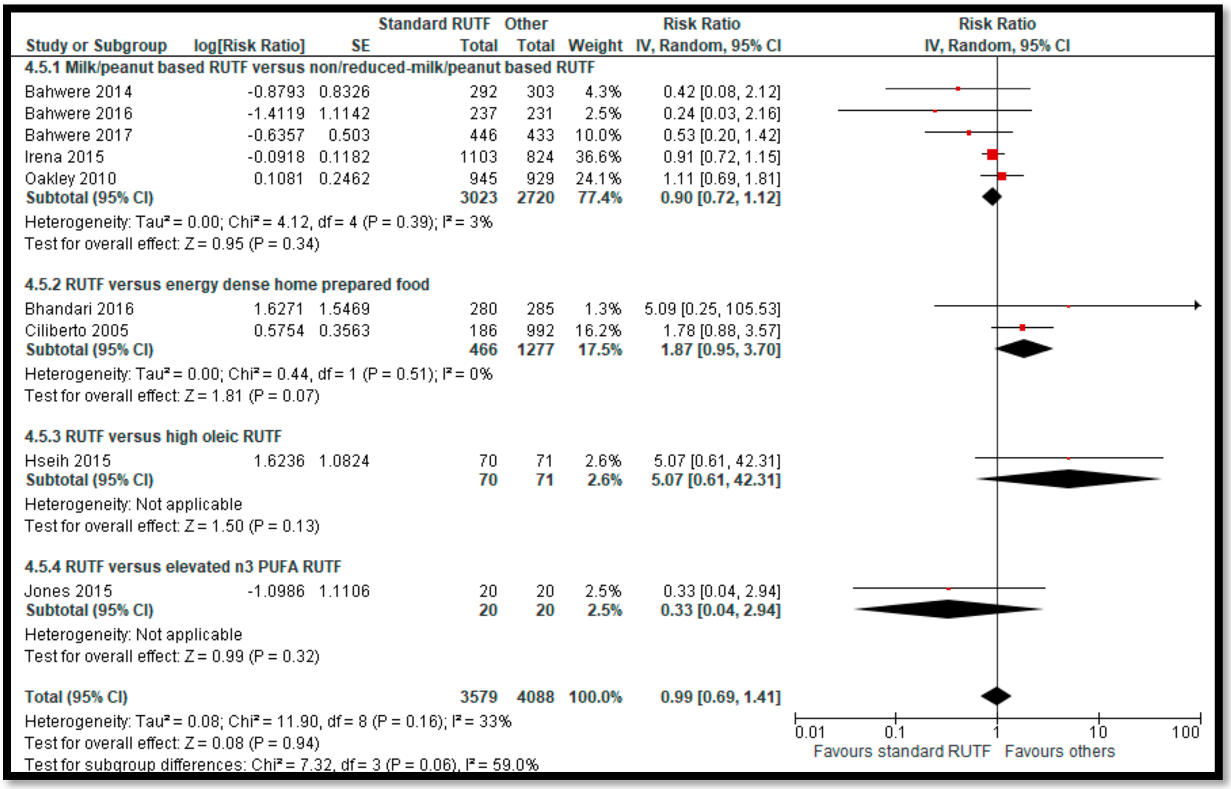

Study or Subgroup	log[Risk Ratio]	SE	Standard RUTF Total	Other Total	Weight	Risk Ratio IV, Random, 95% CI
**4.5.1 Milk/peanut based RUTF versus non/reduced-milk/peanut based RUTF**						
Bahwere 2014	-0.8793	0.8326	292	303	4.3%	0.42 [0.08, 2.12]
Bahwere 2016	-1.4119	1.1142	237	231	2.5%	0.24 [0.03, 2.16]
Bahwere 2017	-0.6357	0.503	446	433	10.0%	0.53 [0.20, 1.42]
Irena 2015	-0.0918	0.1182	1103	824	36.6%	0.91 [0.72, 1.15]
Oakley 2010	0.1081	0.2462	945	929	24.1%	1.11 [0.69, 1.81]
**Subtotal (95% CI)**			**3023**	**2720**	**77.4%**	**0.90 [0.72, 1.12]**
Heterogeneity: Tau² = 0.00; Chi² = 4.12, df = 4 (P = 0.39); I² = 3%						
Test for overall effect: Z = 0.95 (P = 0.34)						
**4.5.2 RUTF versus energy dense home prepared food**						
Bhandari 2016	1.6271	1.5469	280	285	1.3%	5.09 [0.25, 105.53]
Ciliberto 2005	0.5754	0.3563	186	992	16.2%	1.78 [0.88, 3.57]
**Subtotal (95% CI)**			**466**	**1277**	**17.5%**	**1.87 [0.95, 3.70]**
Heterogeneity: Tau² = 0.00; Chi² = 0.44, df = 1 (P = 0.51); I² = 0%						
Test for overall effect: Z = 1.81 (P = 0.07)						
**4.5.3 RUTF versus high oleic RUTF**						
Hseih 2015	1.6236	1.0824	70	71	2.6%	5.07 [0.61, 42.31]
**Subtotal (95% CI)**			**70**	**71**	**2.6%**	**5.07 [0.61, 42.31]**
Heterogeneity: Not applicable						
Test for overall effect: Z = 1.50 (P = 0.13)						
**4.5.4 RUTF versus elevated n3 PUFA RUTF**						
Jones 2015	-1.0986	1.1106	20	20	2.5%	0.33 [0.04, 2.94]
**Subtotal (95% CI)**			**20**	**20**	**2.5%**	**0.33 [0.04, 2.94]**
Heterogeneity: Not applicable						
Test for overall effect: Z = 0.99 (P = 0.32)						
**Total (95% CI)**			**3579**	**4088**	**100.0%**	**0.99 [0.69, 1.41]**

Heterogeneity: Tau² = 0.08; Chi² = 11.90, df = 8 (P = 0.16); I² = 33%
Test for overall effect: Z = 0.08 (P = 0.94)
Test for subgroup differences: Chi² = 7.32, df = 3 (P = 0.06), I² = 59.0%

Figure 12: Forest plot for the impact of RUTF compared to other foods on Height/Length Gain

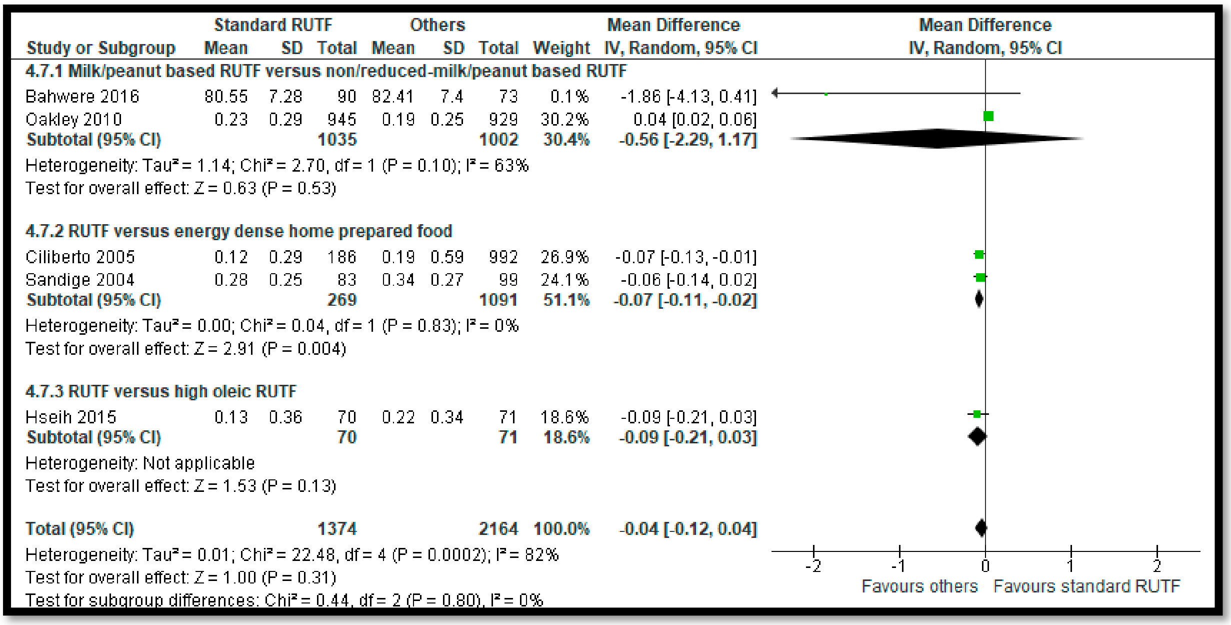

Figure 13: Forest plot for the impact of RUTF compared to other foods on MUAC Gain

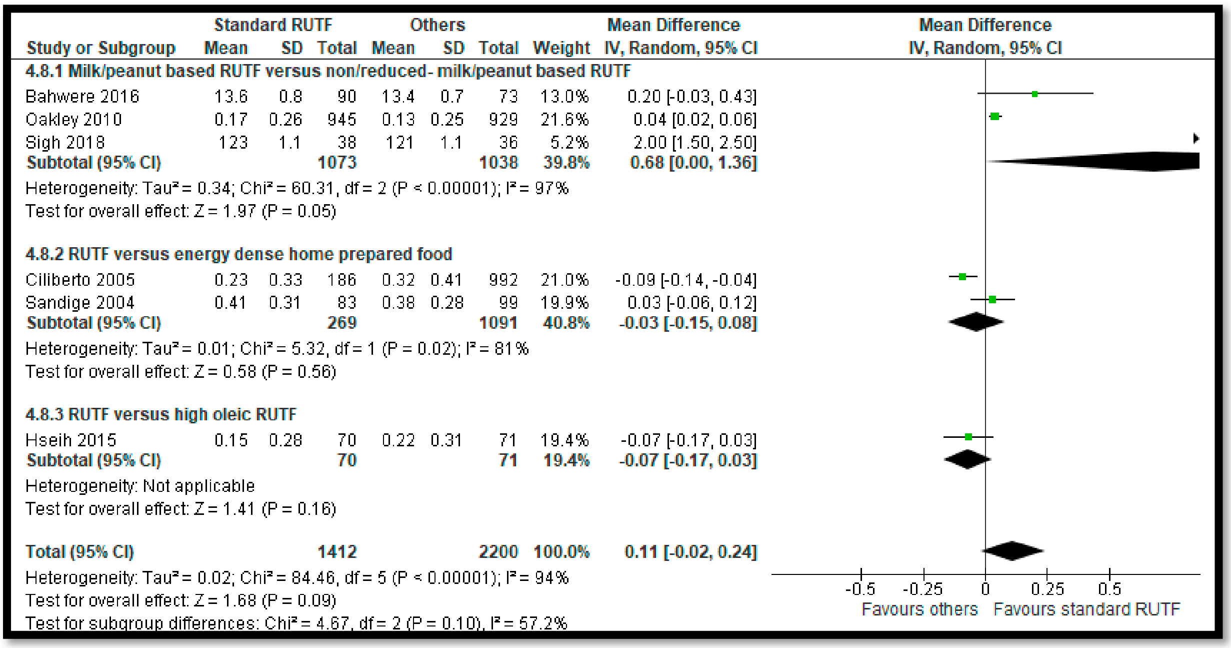

Figure 14: Forest plot for the impact of RUTF compared to other foods on Time to Recovery

Study or Subgroup	Standard RUTF Mean	Standard RUTF SD	Standard RUTF Total	Others Mean	Others SD	Others Total	Weight	Mean Difference IV, Random, 95% CI
**4.9.1 RUTF versus F100**								
Diop 2003	13.4	3.92	35	17.3	5.12	35	42.7%	-3.90 [-6.04, -1.76]
**Subtotal (95% CI)**			**35**			**35**	**42.7%**	**-3.90 [-6.04, -1.76]**
Heterogeneity: Not applicable								
Test for overall effect: Z = 3.58 (P = 0.0003)								
**4.9.2 RUTF versus energy dense home prepared food**								
Bhandari 2016	5.91	4.03	280	7.12	4.54	285	57.3%	-1.21 [-1.92, -0.50]
**Subtotal (95% CI)**			**280**			**285**	**57.3%**	**-1.21 [-1.92, -0.50]**
Heterogeneity: Not applicable								
Test for overall effect: Z = 3.35 (P = 0.0008)								
**Total (95% CI)**			**315**			**320**	**100.0%**	**-2.36 [-4.97, 0.25]**
Heterogeneity: Tau² = 2.96; Chi² = 5.49, df = 1 (P = 0.02); I² = 82%								
Test for overall effect: Z = 1.77 (P = 0.08)								
Test for subgroup differences: Chi² = 5.49, df = 1 (P = 0.02), I² = 81.8%								

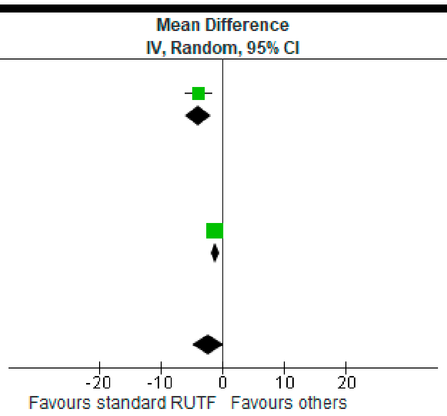

Figure 15: Forest plot for the impact of RUTF compared to other foods on Adverse Events

Study or Subgroup	Standard RUTF Events	Standard RUTF Total	Others Events	Others Total	Weight	Risk Ratio M-H, Random, 95% CI
**4.10.1 Cough/ALRI**						
Bhandari 2016	24	280	38	285	11.3%	0.64 [0.40, 1.04]
Bahwere 2014	43	250	33	278	13.1%	1.45 [0.95, 2.21]
**Subtotal (95% CI)**		**530**		**563**	**24.5%**	**0.97 [0.44, 2.16]**
Total events	67		71			
Heterogeneity: Tau² = 0.28; Chi² = 6.19, df = 1 (P = 0.01); I² = 84%						
Test for overall effect: Z = 0.06 (P = 0.95)						
**4.10.2 Diarrhoea**						
Bhandari 2016	92	280	101	285	20.2%	0.93 [0.74, 1.17]
Jones 2015	7	20	7	20	5.2%	1.00 [0.43, 2.33]
Bahwere 2014	49	258	44	291	14.8%	1.26 [0.87, 1.82]
**Subtotal (95% CI)**		**558**		**596**	**40.2%**	**1.01 [0.83, 1.22]**
Total events	148		152			
Heterogeneity: Tau² = 0.00; Chi² = 1.88, df = 2 (P = 0.39); I² = 0%						
Test for overall effect: Z = 0.08 (P = 0.94)						
**4.10.3 Fever**						
Bhandari 2016	142	280	162	285	23.1%	0.89 [0.77, 1.04]
Bahwere 2014	43	253	27	278	12.2%	1.75 [1.12, 2.74]
**Subtotal (95% CI)**		**533**		**563**	**35.3%**	**1.21 [0.61, 2.39]**
Total events	185		189			
Heterogeneity: Tau² = 0.21; Chi² = 8.25, df = 1 (P = 0.004); I² = 88%						
Test for overall effect: Z = 0.55 (P = 0.58)						
**Total (95% CI)**		**1621**		**1722**	**100.0%**	**1.06 [0.85, 1.32]**
Total events	400		412			
Heterogeneity: Tau² = 0.05; Chi² = 16.38, df = 6 (P = 0.01); I² = 63%						
Test for overall effect: Z = 0.54 (P = 0.59)						
Test for subgroup differences: Chi² = 0.27, df = 2 (P = 0.87), I² = 0%						

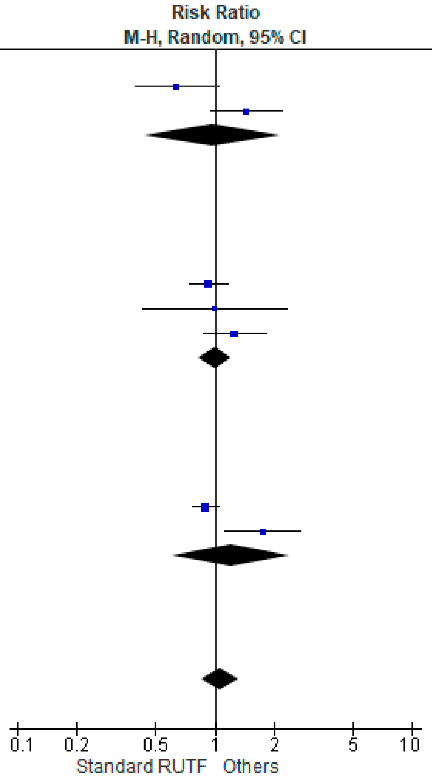

Figure 16: Forest plot for the impact of RUTF compared to other foods on Hospitalisation

Study or Subgroup	Standard RUTF Events	Standard RUTF Total	Control Events	Control Total	Weight	Risk Ratio M-H, Random, 95% CI
Bhandari 2016	30	280	30	285	43.0%	1.02 [0.63, 1.64]
Jones 2015	5	20	4	20	16.9%	1.25 [0.39, 3.99]
Oakley 2010	20	945	39	929	40.1%	0.50 [0.30, 0.86]
**Total (95% CI)**		**1245**		**1234**	**100.0%**	**0.80 [0.46, 1.39]**
Total events	55		73			
Heterogeneity: Tau² = 0.13; Chi² = 4.45, df = 2 (P = 0.11); I² = 55%						
Test for overall effect: Z = 0.80 (P = 0.42)						

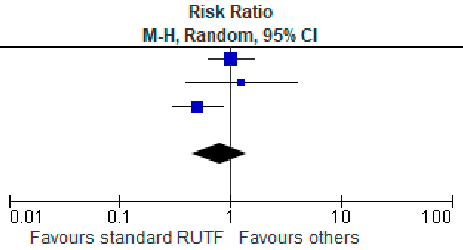

Comparison 5: RUSF for MAM compared to standard diet, or FBF or other locally produced foods

Figure 17: Forest plot for the impact of RUSF for MAM compared to other foods on Recovery

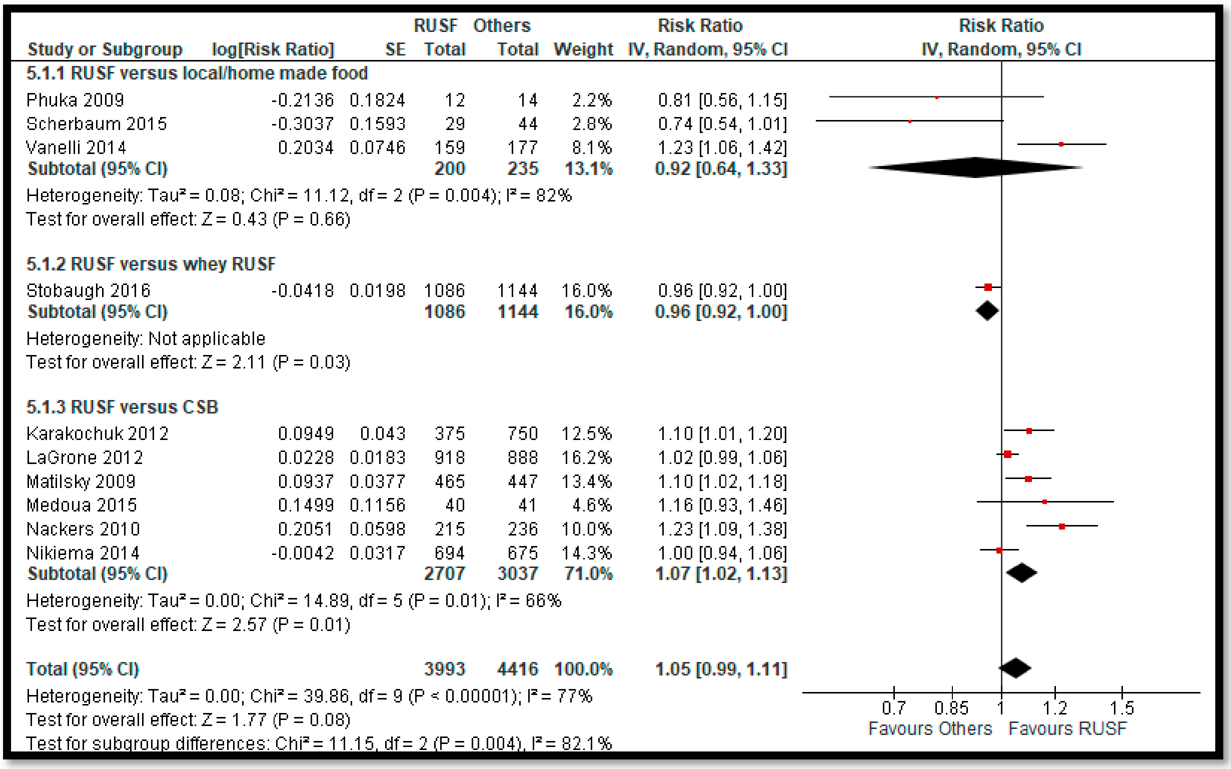

Figure 18: Forest plot for the impact of RUSF for MAM compared to other foods on Weight Gain

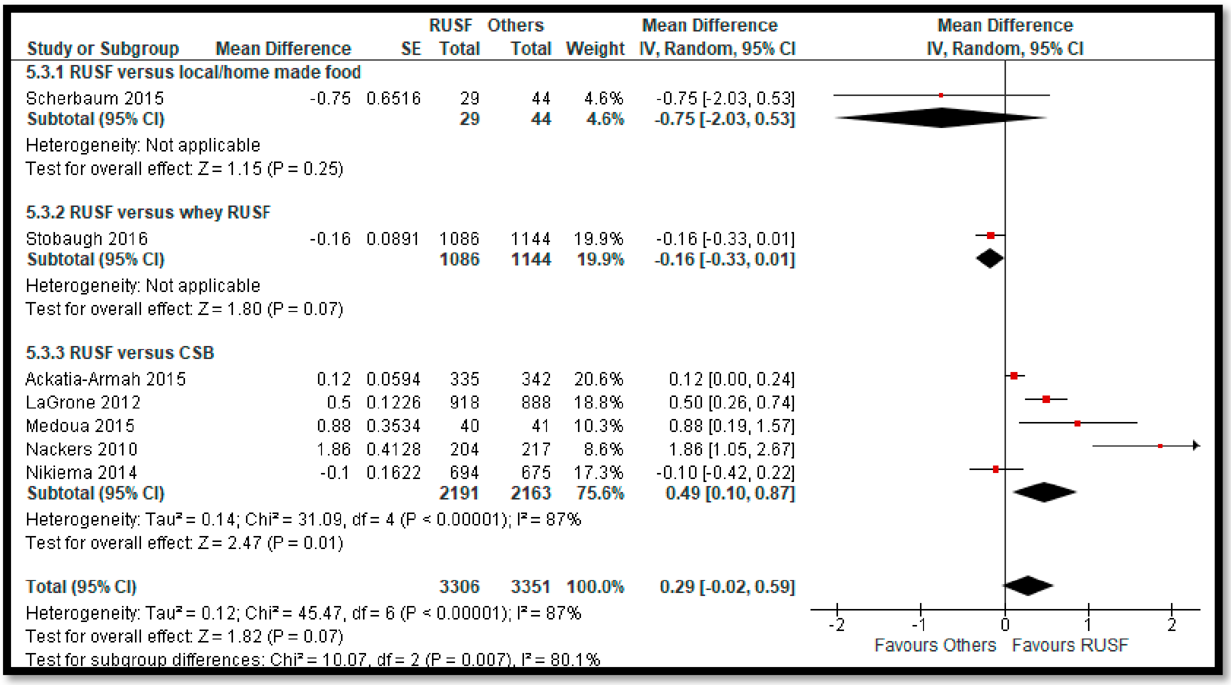

Figure 19: Forest plot for the impact of RUSF for MAM compared to other foods on Mortality

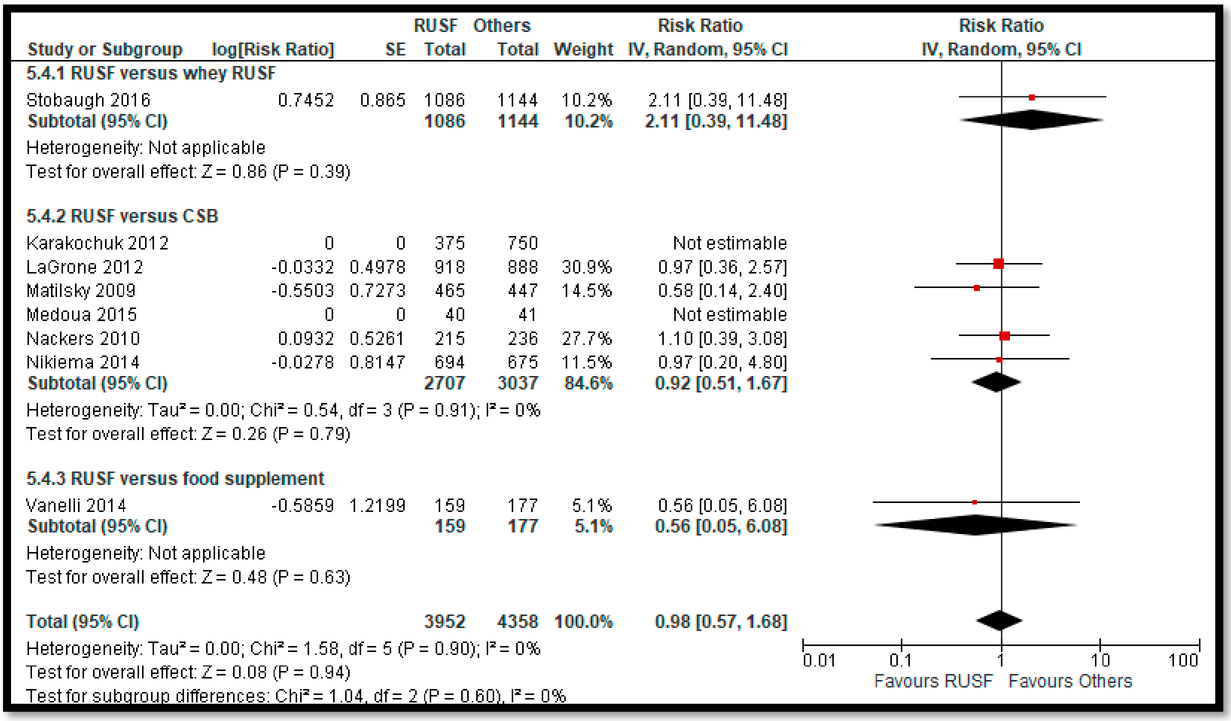

Figure 20: Forest plot for the impact of RUSF for MAM compared to other foods on Length/Height Gain

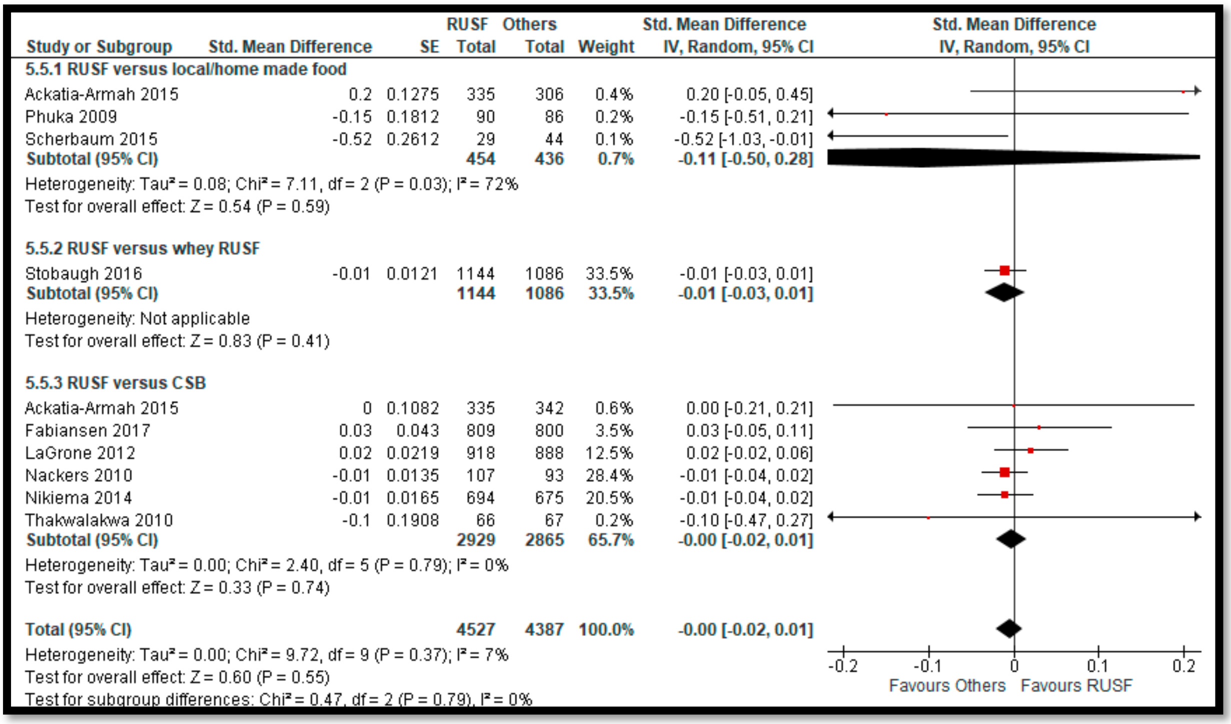

Figure 21: Forest plot for the impact of RUSF for MAM compared to other foods on MUAC

Study or Subgroup	Std. Mean Difference	SE	RUSF Total	Others Total	Weight	Std. Mean Difference IV, Random, 95% CI
**5.7.1 RUSF versus local/home made food**						
Ackatia-Armah 2015	0.3	0.0722	335	306	4.9%	0.30 [0.16, 0.44]
Phuka 2009	0.1	0.1206	90	86	2.0%	0.10 [-0.14, 0.34]
**Subtotal (95% CI)**			**425**	**392**	**6.9%**	**0.22 [0.03, 0.41]**
Heterogeneity: Tau² = 0.01; Chi² = 2.02, df = 1 (P = 0.15); I² = 51%						
Test for overall effect: Z = 2.30 (P = 0.02)						
**5.7.2 RUSF versus whey RUSF**						
Stobaugh 2016	0.04	0.0117	1144	1086	21.4%	0.04 [0.02, 0.06]
**Subtotal (95% CI)**			**1144**	**1086**	**21.4%**	**0.04 [0.02, 0.06]**
Heterogeneity: Not applicable						
Test for overall effect: Z = 3.42 (P = 0.0006)						
**5.7.3 RUSF versus CSB**						
Ackatia-Armah 2015	0.2	0.0722	335	342	4.9%	0.20 [0.06, 0.34]
LaGrone 2012	0.08	0.0198	918	888	18.3%	0.08 [0.04, 0.12]
Medoua 2015	0.04	0.0352	40	41	12.4%	0.04 [-0.03, 0.11]
Nackers 2010	0.05	0.0301	162	150	14.2%	0.05 [-0.01, 0.11]
Nikiema 2014	0.04	0.0154	694	675	20.0%	0.04 [0.01, 0.07]
Thakwalakwa 2010	0.3	0.1228	66	67	2.0%	0.30 [0.06, 0.54]
**Subtotal (95% CI)**			**2215**	**2163**	**71.7%**	**0.07 [0.03, 0.10]**
Heterogeneity: Tau² = 0.00; Chi² = 10.68, df = 5 (P = 0.06); I² = 53%						
Test for overall effect: Z = 3.58 (P = 0.0003)						
**Total (95% CI)**			**3784**	**3641**	**100.0%**	**0.08 [0.04, 0.11]**
Heterogeneity: Tau² = 0.00; Chi² = 24.00, df = 8 (P = 0.002); I² = 67%						
Test for overall effect: Z = 4.21 (P < 0.0001)						
Test for subgroup differences: Chi² = 4.77, df = 2 (P = 0.09), I² = 58.1%						

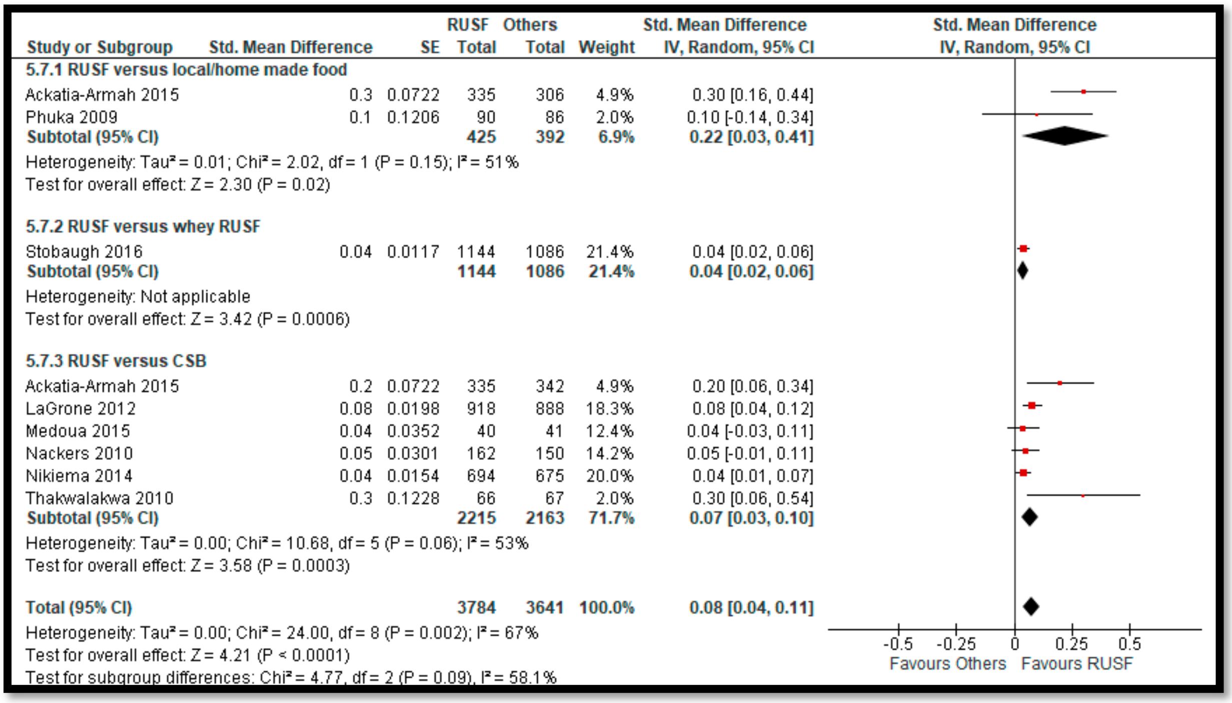

Figure 22: Forest plot for the impact of RUSF for MAM compared to other foods on Time to Recovery

Study or Subgroup	Mean Difference	SE	RUSF Total	Others Total	Weight	Mean Difference IV, Random, 95% CI
**5.8.1 RUSF versus local/home made food**						
Scherbaum 2015	-14.2	6.0597	18	37	8.4%	-14.20 [-26.08, -2.32]
**Subtotal (95% CI)**			**18**	**37**	**8.4%**	**-14.20 [-26.08, -2.32]**
Heterogeneity: Not applicable						
Test for overall effect: Z = 2.34 (P = 0.02)						
**5.8.2 RUSF versus whey RUSF**						
Stobaugh 2016	-1.1	0.8292	1144	1086	22.6%	-1.10 [-2.73, 0.53]
**Subtotal (95% CI)**			**1144**	**1086**	**22.6%**	**-1.10 [-2.73, 0.53]**
Heterogeneity: Not applicable						
Test for overall effect: Z = 1.33 (P = 0.18)						
**5.8.3 RUSF versus CSB**						
LaGrone 2012	-2.3	0.7681	918	888	22.7%	-2.30 [-3.81, -0.79]
Medoua 2015	-6.99	0.3724	40	41	23.2%	-6.99 [-7.72, -6.26]
Nikiema 2014	1	0.3505	694	675	23.2%	1.00 [0.31, 1.69]
**Subtotal (95% CI)**			**1652**	**1604**	**69.0%**	**-2.77 [-8.39, 2.86]**
Heterogeneity: Tau² = 24.39; Chi² = 244.41, df = 2 (P < 0.00001); I² = 99%						
Test for overall effect: Z = 0.96 (P = 0.33)						
**Total (95% CI)**			**2814**	**2727**	**100.0%**	**-3.35 [-7.68, 0.97]**
Heterogeneity: Tau² = 20.85; Chi² = 251.55, df = 4 (P < 0.00001); I² = 98%						
Test for overall effect: Z = 1.52 (P = 0.13)						
Test for subgroup differences: Chi² = 4.82, df = 2 (P = 0.09), I² = 58.5%						

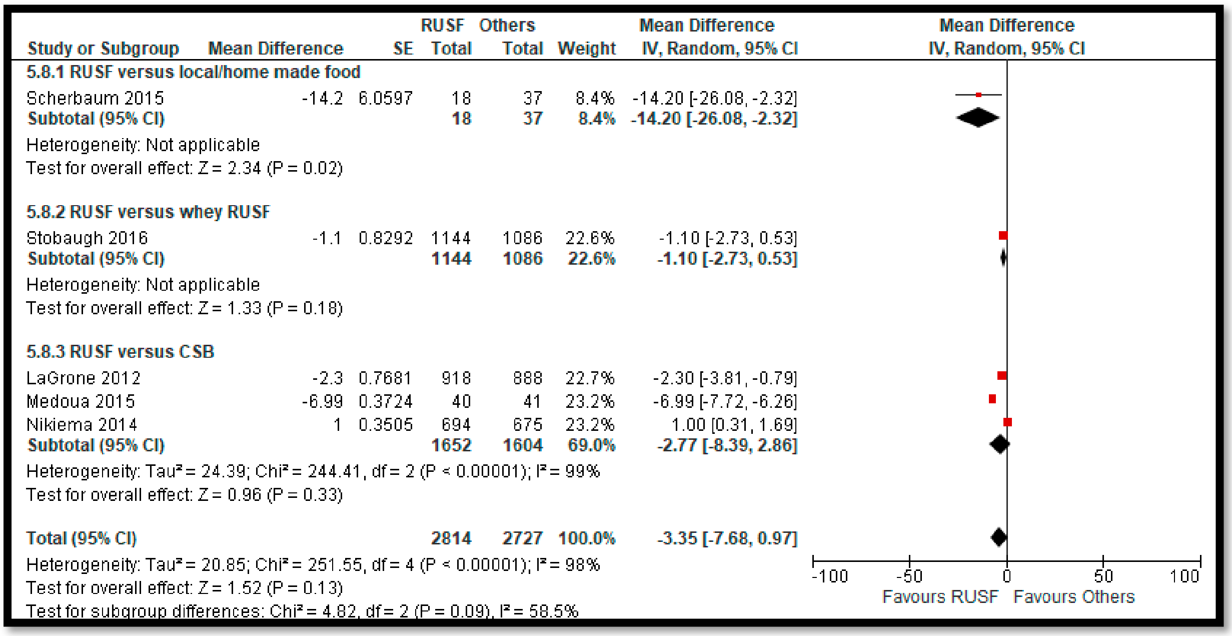

Figure 23: Forest plot for the impact of RUSF for MAM compared to other foods on Moderate Stunting

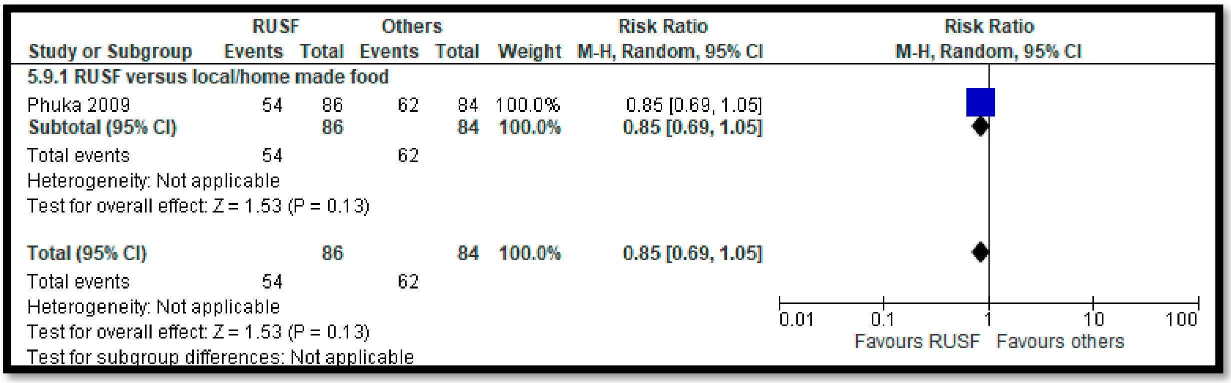

Figure 24: Forest plot for the impact of RUSF for MAM compared to other foods on Moderate Wasting

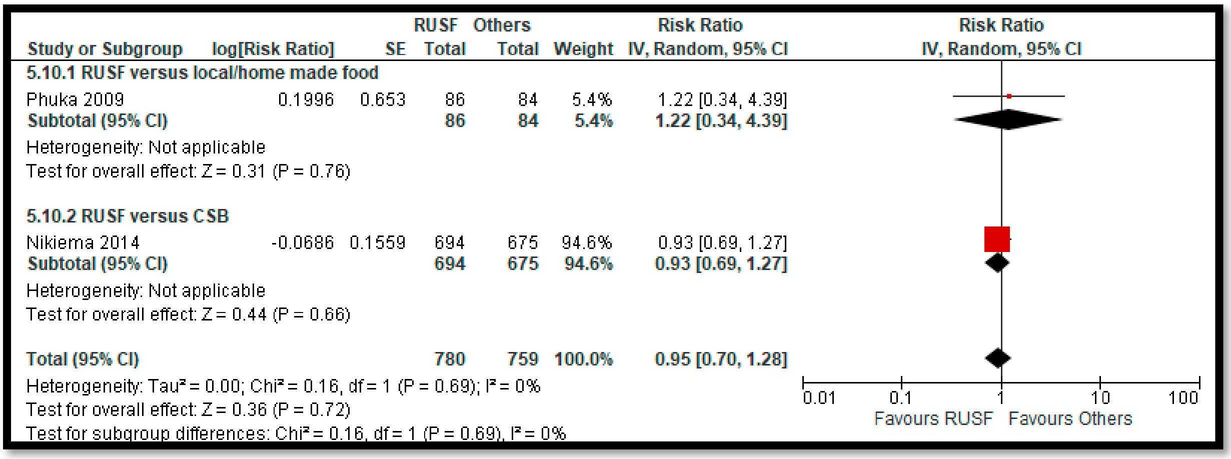

Figure 25: Forest plot for the impact of RUSF for MAM compared to other foods on Severe Wasting

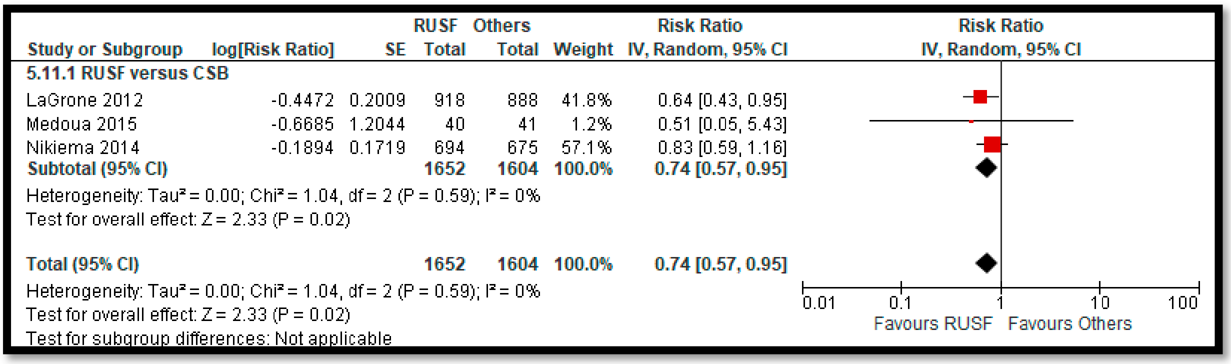

Figure 26: Forest plot for the impact of RUSF for MAM compared to other foods on Moderate Underweight

Study or Subgroup	RUSF Events	RUSF Total	Others Events	Others Total	Weight	Risk Ratio M-H, Random, 95% CI
**5.12.1 RUSF versus local/home made food**						
Phuka 2009	74	86	68	84	100.0%	1.06 [0.93, 1.22]
**Subtotal (95% CI)**		**86**		**84**	**100.0%**	**1.06 [0.93, 1.22]**
Total events	74		68			
Heterogeneity: Not applicable						
Test for overall effect: Z = 0.89 (P = 0.37)						
**Total (95% CI)**		**86**		**84**	**100.0%**	**1.06 [0.93, 1.22]**
Total events	74		68			
Heterogeneity: Not applicable						
Test for overall effect: Z = 0.89 (P = 0.37)						
Test for subgroup differences: Not applicable						

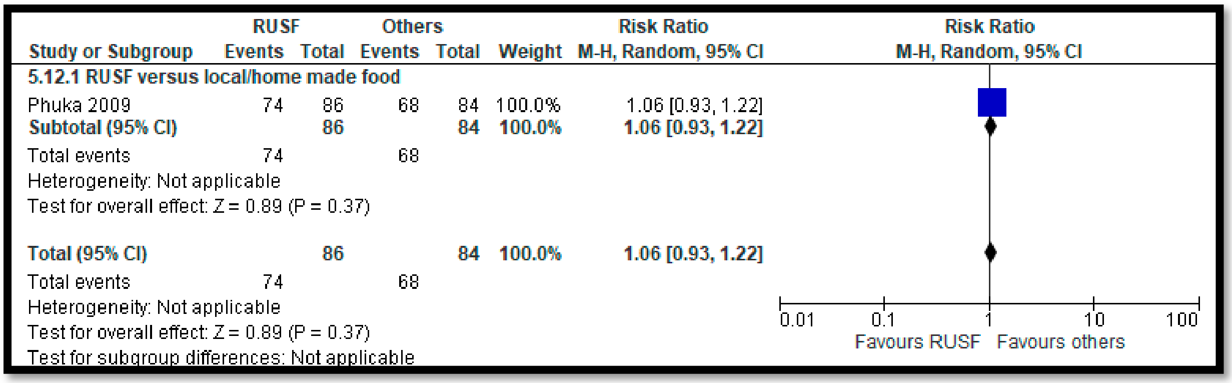

Figure 27: Forest plot for the impact of RUSF for MAM compared to other foods on Adverse Events

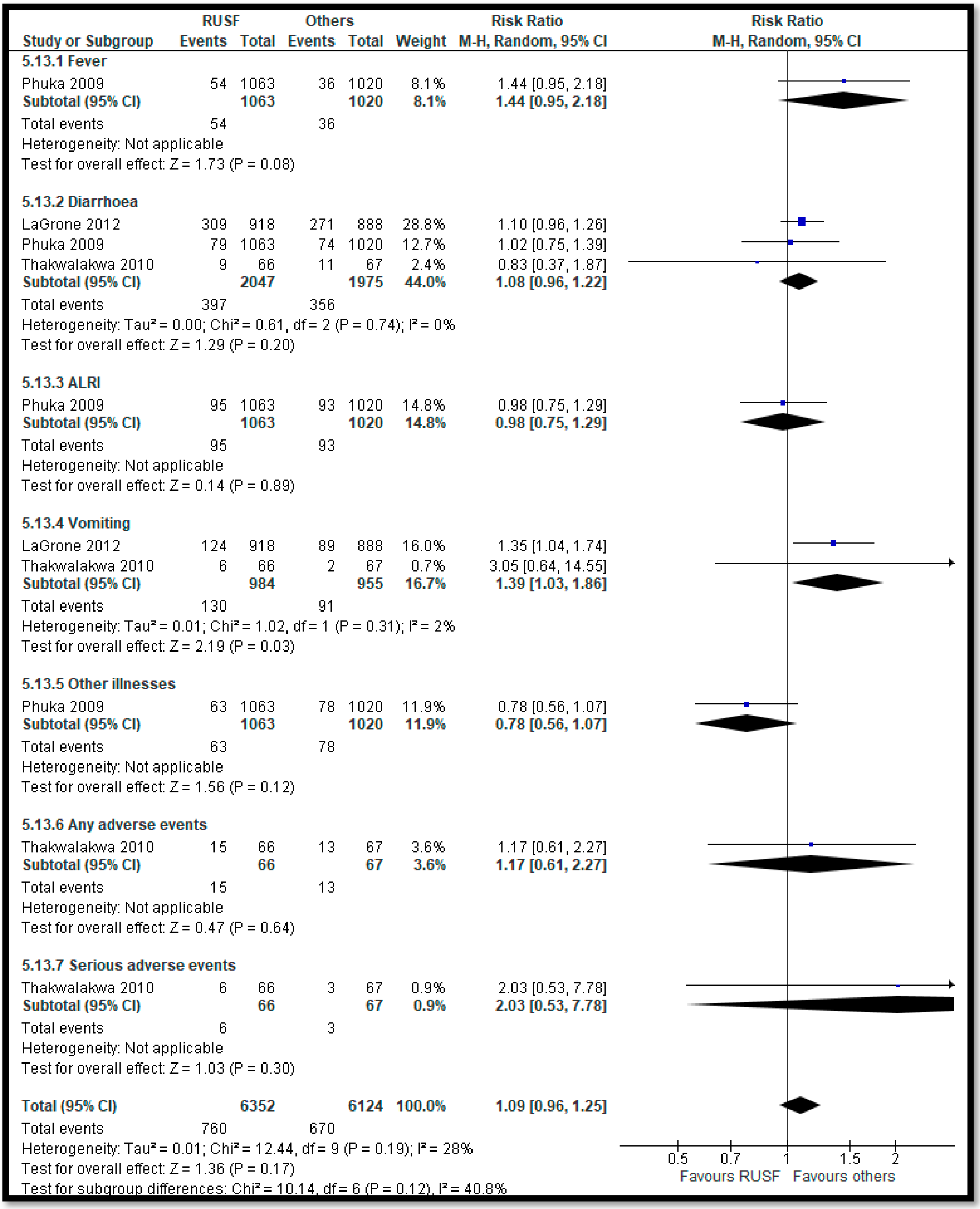

Figure 28: Forest plot for the impact of RUSF for MAM compared to other foods on Hospitalisation

Study or Subgroup	RUSF Events	RUSF Total	Others Events	Others Total	Weight	Risk Ratio M-H, Random, 95% CI
Ackatia-Armah 2015	0	335	1	342	5.8%	0.34 [0.01, 8.32]
Karakochuk 2012	6	375	8	750	32.2%	1.50 [0.52, 4.29]
LaGrone 2012	1	918	0	888	5.8%	2.90 [0.12, 71.14]
Medoua 2015	0	40	0	41		Not estimable
Nackers 2010	20	215	45	236	56.2%	0.49 [0.30, 0.80]
**Total (95% CI)**		**1883**		**2257**	**100.0%**	**0.76 [0.34, 1.70]**
Total events	27		54			

Heterogeneity: Tau² = 0.24; Chi² = 4.65, df = 3 (P = 0.20); I² = 35%
Test for overall effect: Z = 0.67 (P = 0.51)

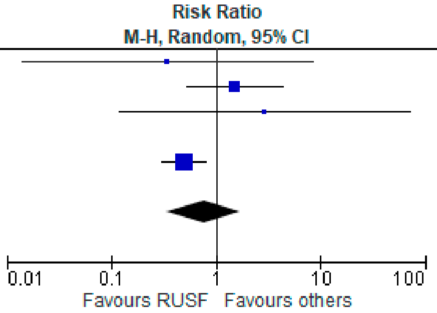

Comparison 6: Prophylactic use of antibiotics in children with uncomplicated SAM compared to no antibiotics

Figure 29: Forest plot for the impact of prophylactic antibiotic compared to no antibiotic on Weight Gain

Study or Subgroup	Mean Difference	SE	Antibiotic Total	No antibiotic Total	Weight	Mean Difference IV, Random, 95% CI
Isanaka 2016	0.9	0.1634	1199	1200	53.4%	0.90 [0.58, 1.22]
Manary 2012 (1)	0.551	0.187	1780	873	46.6%	0.55 [0.18, 0.92]
**Total (95% CI)**			**2979**	**2073**	**100.0%**	**0.74 [0.40, 1.08]**

Heterogeneity: Tau² = 0.03; Chi² = 1.98, df = 1 (P = 0.16); I² = 49%
Test for overall effect: Z = 4.24 (P < 0.0001)

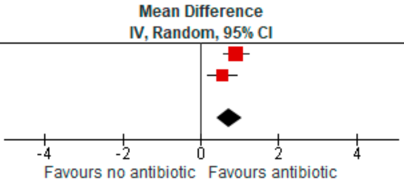

Footnotes
(1) Cefdinir

Figure 30: Forest plot for the impact of prophylactic antibiotic compared to no antibiotic on MUAC Gain

Study or Subgroup	Mean Difference	SE	Antibiotic Total	No antibiotic Total	Weight	Mean Difference IV, Random, 95% CI
Isanaka 2016	0.06	0.0112	1199	1200	70.0%	0.06 [0.04, 0.08]
Manary 2012	0.055	0.0171	1766	866	30.0%	0.06 [0.02, 0.09]
**Total (95% CI)**			**2965**	**2066**	**100.0%**	**0.06 [0.04, 0.08]**

Heterogeneity: Tau² = 0.00; Chi² = 0.06, df = 1 (P = 0.81); I² = 0%
Test for overall effect: Z = 6.24 (P < 0.00001)

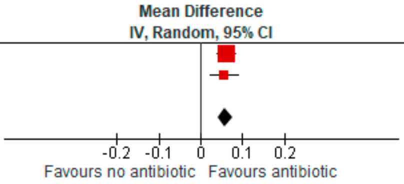

Figure 31: Forest plot for the impact of prophylactic antibiotic compared to no antibiotic on Length Gain

Study or Subgroup	Mean Difference	SE	Antibiotic Total	No antibiotic Total	Weight	Mean Difference IV, Random, 95% CI
Isanaka 2016	0	0.0063	1199	1200	66.2%	0.00 [-0.01, 0.01]
Manary 2012	0.03	0.0182	1780	873	33.8%	0.03 [-0.01, 0.07]
**Total (95% CI)**			**2979**	**2073**	**100.0%**	**0.01 [-0.02, 0.04]**

Heterogeneity: Tau² = 0.00; Chi² = 2.43, df = 1 (P = 0.12); I² = 59%
Test for overall effect: Z = 0.71 (P = 0.47)

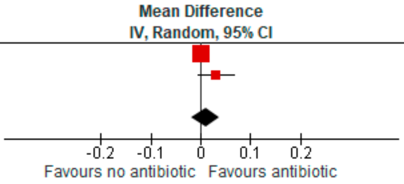

Figure 32: Forest plot for the impact of prophylactic antibiotic compared to no antibiotic on Time to Recovery

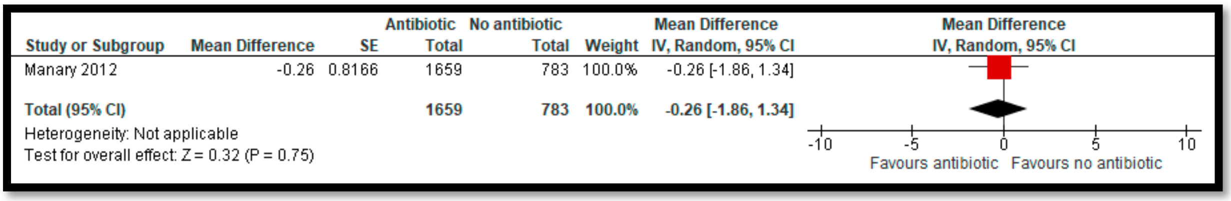

Study or Subgroup	Mean Difference	SE	Antibiotic Total	No antibiotic Total	Weight	Mean Difference IV, Random, 95% CI
Manary 2012	-0.26	0.8166	1659	783	100.0%	-0.26 [-1.86, 1.34]
**Total (95% CI)**			**1659**	**783**	**100.0%**	**-0.26 [-1.86, 1.34]**

Heterogeneity: Not applicable
Test for overall effect: Z = 0.32 (P = 0.75)

Figure 33: Forest plot for the impact of prophylactic antibiotic compared to no antibiotic on Adverse Events

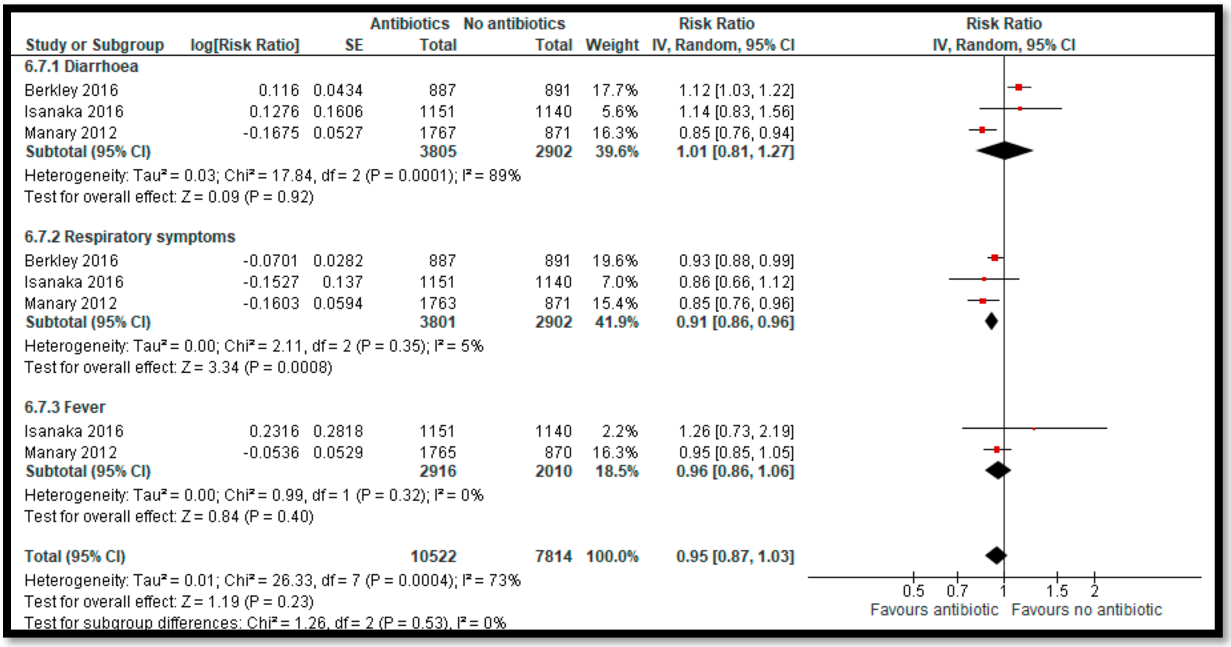

Study or Subgroup	log[Risk Ratio]	SE	Antibiotics Total	No antibiotics Total	Weight	Risk Ratio IV, Random, 95% CI
**6.7.1 Diarrhoea**						
Berkley 2016	0.116	0.0434	887	891	17.7%	1.12 [1.03, 1.22]
Isanaka 2016	0.1276	0.1606	1151	1140	5.6%	1.14 [0.83, 1.56]
Manary 2012	-0.1675	0.0527	1767	871	16.3%	0.85 [0.76, 0.94]
**Subtotal (95% CI)**			**3805**	**2902**	**39.6%**	**1.01 [0.81, 1.27]**
Heterogeneity: Tau² = 0.03; Chi² = 17.84, df = 2 (P = 0.0001); I² = 89%						
Test for overall effect: Z = 0.09 (P = 0.92)						
**6.7.2 Respiratory symptoms**						
Berkley 2016	-0.0701	0.0282	887	891	19.6%	0.93 [0.88, 0.99]
Isanaka 2016	-0.1527	0.137	1151	1140	7.0%	0.86 [0.66, 1.12]
Manary 2012	-0.1603	0.0594	1763	871	15.4%	0.85 [0.76, 0.96]
**Subtotal (95% CI)**			**3801**	**2902**	**41.9%**	**0.91 [0.86, 0.96]**
Heterogeneity: Tau² = 0.00; Chi² = 2.11, df = 2 (P = 0.35); I² = 5%						
Test for overall effect: Z = 3.34 (P = 0.0008)						
**6.7.3 Fever**						
Isanaka 2016	0.2316	0.2818	1151	1140	2.2%	1.26 [0.73, 2.19]
Manary 2012	-0.0536	0.0529	1765	870	16.3%	0.95 [0.85, 1.05]
**Subtotal (95% CI)**			**2916**	**2010**	**18.5%**	**0.96 [0.86, 1.06]**
Heterogeneity: Tau² = 0.00; Chi² = 0.99, df = 1 (P = 0.32); I² = 0%						
Test for overall effect: Z = 0.84 (P = 0.40)						
**Total (95% CI)**			**10522**	**7814**	**100.0%**	**0.95 [0.87, 1.03]**

Heterogeneity: Tau² = 0.01; Chi² = 26.33, df = 7 (P = 0.0004); I² = 73%
Test for overall effect: Z = 1.19 (P = 0.23)
Test for subgroup differences: Chi² = 1.26, df = 2 (P = 0.53), I² = 0%

Figure 34: Forest plot for the impact of prophylactic antibiotic compared to no antibiotic on Hospitalisation

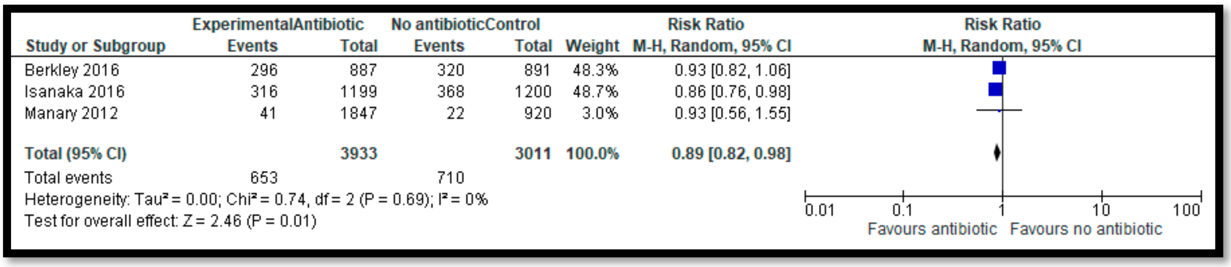

Study or Subgroup	ExperimentalAntibiotic Events	ExperimentalAntibiotic Total	No antibioticControl Events	No antibioticControl Total	Weight	Risk Ratio M-H, Random, 95% CI
Berkley 2016	296	887	320	891	48.3%	0.93 [0.82, 1.06]
Isanaka 2016	316	1199	368	1200	48.7%	0.86 [0.76, 0.98]
Manary 2012	41	1847	22	920	3.0%	0.93 [0.56, 1.55]
**Total (95% CI)**		**3933**		**3011**	**100.0%**	**0.89 [0.82, 0.98]**
Total events	653		710			

Heterogeneity: Tau² = 0.00; Chi² = 0.74, df = 2 (P = 0.69); I² = 0%
Test for overall effect: Z = 2.46 (P = 0.01)

Comparison 7: Vitamin A supplementation in the management of SAM and MAM with various doses and frequency of administration

Figure 35: Forest plot for the impact of vitamin A supplementation on Weight Change

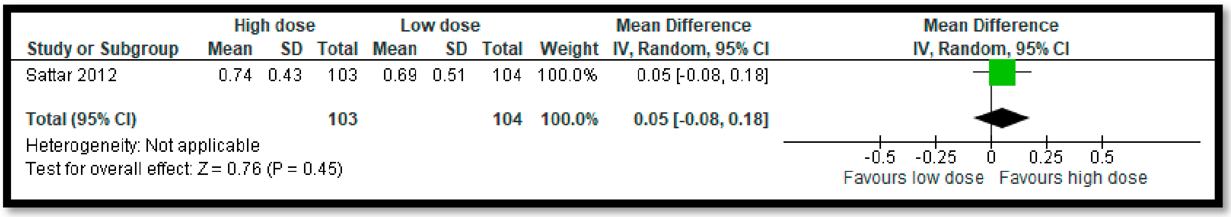

Study or Subgroup	High dose Mean	High dose SD	High dose Total	Low dose Mean	Low dose SD	Low dose Total	Weight	Mean Difference IV, Random, 95% CI
Sattar 2012	0.74	0.43	103	0.69	0.51	104	100.0%	0.05 [-0.08, 0.18]
**Total (95% CI)**			**103**			**104**	**100.0%**	**0.05 [-0.08, 0.18]**

Heterogeneity: Not applicable
Test for overall effect: Z = 0.76 (P = 0.45)

Figure 36: Forest plot for the impact of vitamin A supplementation on Mortality

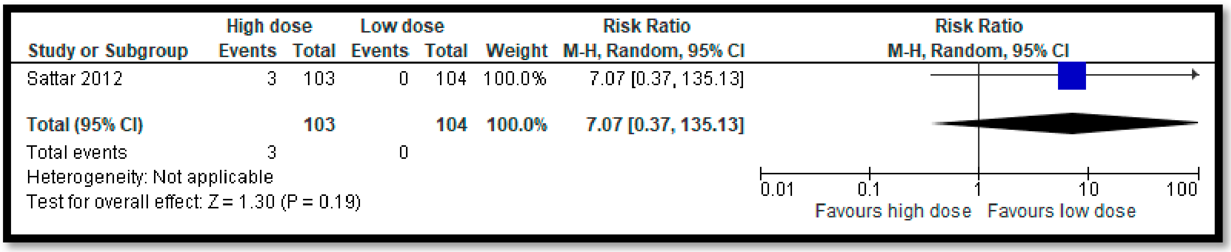

Figure 37: Forest plot for the impact of vitamin A supplementation on Height Change

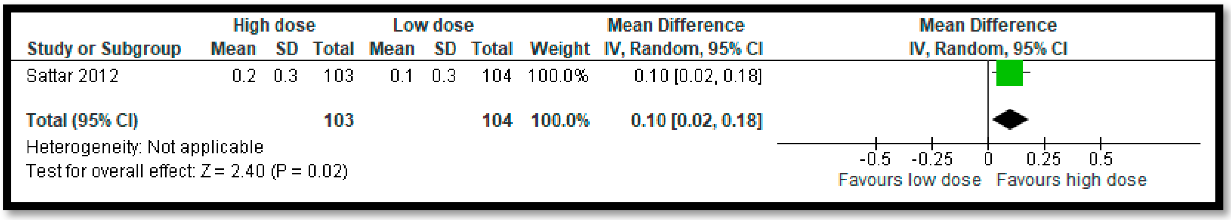

Figure 38: Forest plot for the impact of vitamin A supplementation on MUAC Change

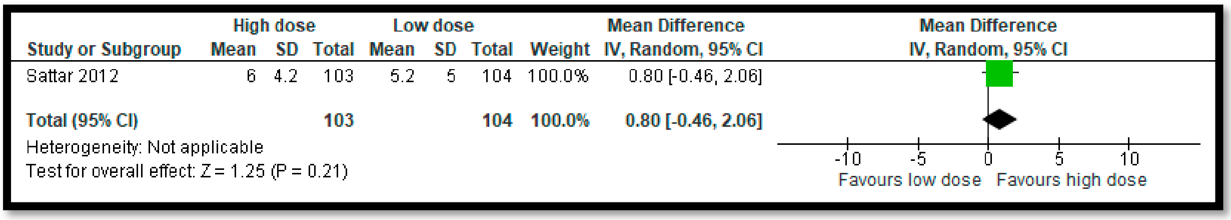

Figure 39: Forest plot for the impact of vitamin A supplementation on Adverse Events

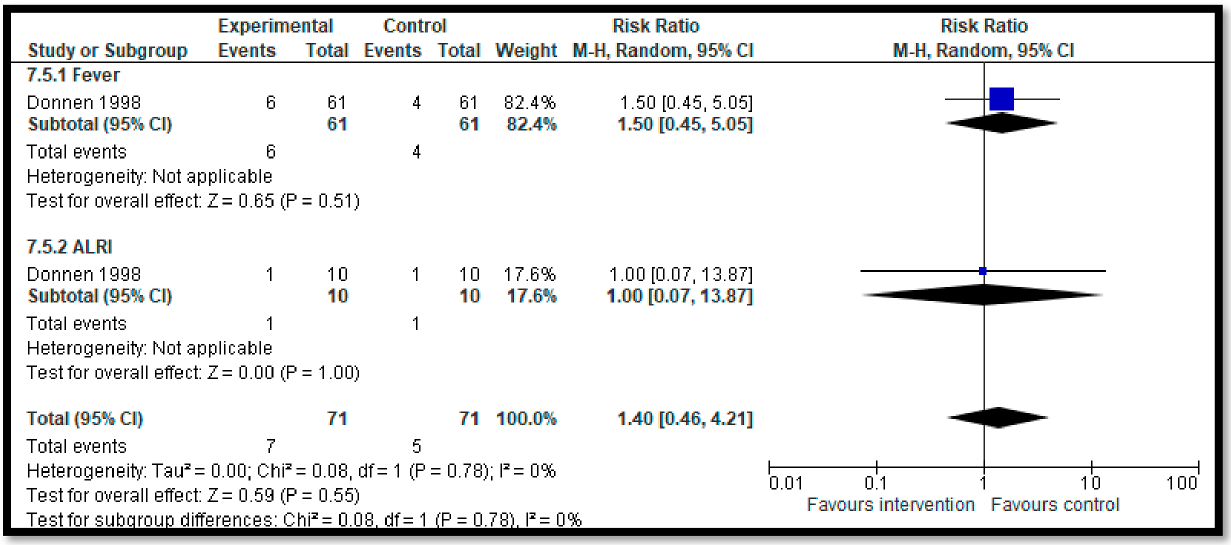

1. Agha, S., *Supplementary feeding of malnourished children in northern Iraq.* 2004.

2. Aguayo, V.M., et al., *Community management of acute malnutrition (CMAM) programme in P akistan effectively treats children with uncomplicated severe wasting.* Maternal & child nutrition, 2018. **14**: p. e12623.
3. Ahmed, T., et al., *Mortality in severely malnourished children with diarrhoea and use of a standardised management protocol.* The Lancet, 1999. **353**(9168): p. 1919-1922.
4. Ashworth, A., et al., *WHO guidelines for management of severe malnutrition in rural South African hospitals: effect on case fatality and the influence of operational factors.* The Lancet, 2004. **363**(9415): p. 1110-1115.
5. Bachou, H., et al., *Reduction of unnecessary transfusion and intravenous fluids in severely malnourished children is not enough to reduce mortality.* Annals of tropical paediatrics, 2008. **28**(1): p. 23-33.
6. Badaloo, A., et al., *Dietary protein, growth and urea kinetics in severely malnourished children and during recovery.* The Journal of nutrition, 1999. **129**(5): p. 969-979.
7. Baker, R., et al., *Successful use of a soya-maize mixture in the treatment of kwashiorkor.* S Afr Med J, 1978. **53**(17): p. 674-677.
8. Bhandari, N., et al., *Food supplementation with encouragement to feed it to infants from 4 to 12 months of age has a small impact on weight gain.* The Journal of nutrition, 2001. **131**(7): p. 1946-1951.
9. Burza, S., et al., *Seasonal effect and long-term nutritional status following exit from a community-based management of severe acute malnutrition program in Bihar, India.* European journal of clinical nutrition, 2016. **70**(4): p. 437.
10. Donnen, P., et al., *Effect of daily low dose of vitamin A compared with single high dose on morbidity and mortality of hospitalized mainly malnourished children in senegal: a randomized controlled clinical trial.* European journal of clinical nutrition, 2007. **61**(12): p. 1393.
11. Dubray, C., et al., *Treatment of severe malnutrition with 2-day intramuscular ceftriaxone vs 5-day amoxicillin.* Annals of tropical paediatrics, 2008. **28**(1): p. 13-22.
12. Javan, R., et al., *Effectiveness of supplementary blended flour based on chickpea and cereals for the treatment of infants with moderate acute malnutrition in Iran: A randomized clinical trial.* Electronic physician, 2017. **9**(12): p. 6078.
13. Linneman, Z., et al., *A large-scale operational study of home-based therapy with ready-to-use therapeutic food in childhood malnutrition in Malawi.* Maternal & child nutrition, 2007. **3**(3): p. 206-215.
14. Nagar, R.P., T. Nagar, and B.D. Gupta, *Treatment outcome in patients with severe acute malnutrition managed with protocolised care at malnutrition treatment corner in Rajasthan, India: a prospective observational study (quasi-experimental).* Int J Res Med Sci, 2016. **4**(1): p. 238-45.
15. Roy, S.K., et al., *Intensive nutrition education with or without supplementary feeding improves the nutritional status of moderately-malnourished children in Bangladesh.* Journal of Health, Population and Nutrition, 2005: p. 320-330.
16. Simpore, J., et al., *Nutrition rehabilitation of undernourished children utilizing Spiruline and Misola.* Nutrition journal, 2006. **5**(1): p. 3.
17. Zongo, U., et al., *Nutritional and clinical rehabilitation of severely malnourished children with Moringa oleifera Lam. leaf powder in Ouagadougou (Burkina Faso).* Food and nutrition sciences, 2013. **4**(09): p. 991.
